# Disrupted thalamic functional hierarchy on resting-state fMRI in idiopathic generalized epilepsy

**DOI:** 10.1186/s41747-026-00788-x

**Published:** 2026-09-15

**Authors:** Junxia Chen, Xueqing Sun, Kai Yu, Zhihuan Yang, Hechun Li, Zeling Yang, Yuecong Chen, Jianfu Li, Roberto Rodriguez-Labrada, Dezhong Yao, Sisi Jiang, Cheng Luo

**Affiliations:** 1 The Clinical Hospital of Chengdu Brain Science Institute, MOE Key Lab for Neuroinformation, School of Life Science and Technology, University of Electronic Science and Technology of China, Chengdu, P. R. China; 2 China-Cuba Belt and Road Joint Laboratory on Neurotechnology and Brain-Apparatus Communication, University of Electronic Science and Technology of China, Chengdu, P. R. China; 3 Research Unit of NeuroInformation, Chinese Academy of Medical Sciences, Chengdu, P. R. China; 4 High-Field Magnetic Resonance Brain Imaging Key Laboratory of Sichuan Province, Center for Information in Medicine, University of Electronic Science and Technology of China, Chengdu, P. R. China; 5 Cuban Neuroscience Center, La Habana, Cuba; 6 Department of Radiology, The Fourth People’s Hospital of Chengdu, Chengdu, P. R. China

**Keywords:** Connectome, Epilepsy, Functional neuroimaging, Magnetic resonance imaging, Thalamus

## Abstract

**Objective:**

Abnormal thalamo-cerebral and thalamo-cerebellar interactions are implicated in idiopathic generalized epilepsy (IGE), but their influence on thalamic functional hierarchy remains unclear. This study aimed to characterize thalamic functional hierarchy across varying connectivity strengths in IGE.

**Materials and methods:**

Resting-state functional magnetic resonance imaging (fMRI) data were collected for 144 IGE patients and 222 healthy controls. The voxel-wise thalamo-cerebral and thalamo-cerebellar functional connectivity (FC) was first computed. Connectome gradient mapping was applied separately to anticorrelated and correlated connectivity strengths for both thalamo-cerebral and thalamo-cerebellar pathways. Furthermore, the first three thalamic gradient features were investigated using eccentricity.

**Results:**

In both healthy controls and patients with IGE, the thalamus predominantly coupled with low-order unimodal networks under anticorrelated conditions, and with high-order cognitive networks under correlated conditions. Moreover, connectome-dependent alterations were further observed in the limbic network, attentional network, and cerebellar systems in IGE. Additionally, thalamic gradients in IGE showed marked distributional abnormalities across a broad range of connectivity strengths. Notably, both thalamo-cerebral and thalamo-cerebellar eccentricity revealed shared increases and decreases within motor and cognitive thalamic nuclei, together with additional pathway-specific abnormalities unique to the thalamo-cerebellar system. These dispersion abnormalities were associated with poorer working memory performance in IGE, with three significant thalamic clusters identified (all *p* ≤ 0.008).

**Conclusion:**

These fMRI findings demonstrate disruption of the thalamic functional hierarchy, manifested as nucleus-specific alterations in integration and segregation in IGE. Collectively, this study provides novel insights into thalamic functional abnormalities in epilepsy.

**Key Points:**

- ***Question*** The hierarchical disruption of thalamo-cerebral and thalamo-cerebellar pathways in idiopathic generalized epilepsy and its clinical significance remains unclear.
- ***Findings*** Both pathways show altered functional hierarchy, with eccentricity changes in specific thalamic nuclei linked to slower working memory performance in idiopathic generalized epilepsy.
- ***Relevance statement*** This study revealed nucleus-specific integration-segregation disruptions in IGE that correlated with poorer working memory performance, providing an important window into thalamic dysfunction mechanisms.

**Graphical Abstract:**

**Figure:**
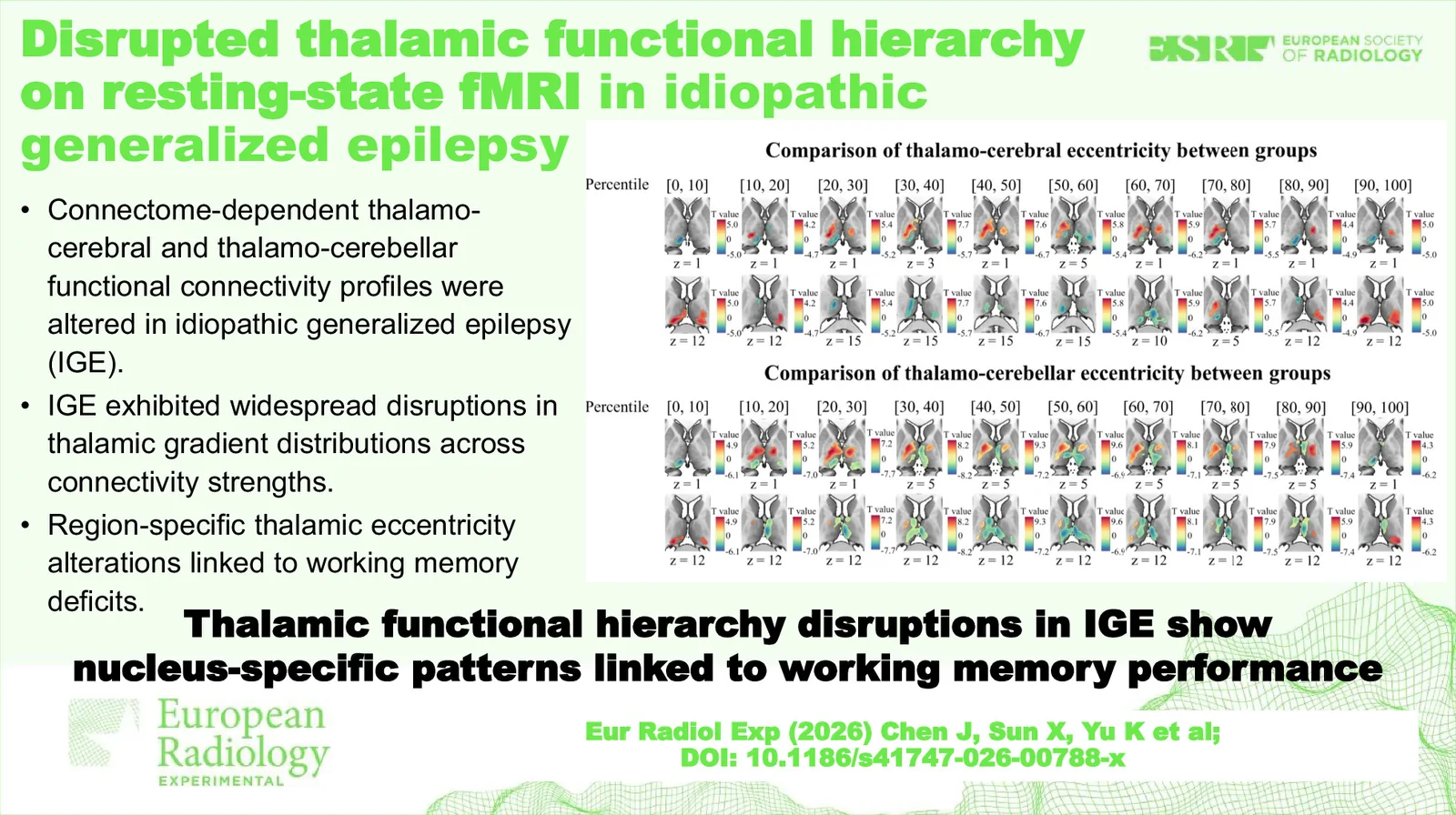

## Background

Idiopathic generalized epilepsy (IGE) with generalized tonic-clonic seizures is characterized by 2.5–5 Hz generalized spike-wave discharges [1]. The thalamo-cortical network is a core pathological substrate and therapeutic target [2, 3]. The thalamus acts as a hub in ictogenesis, synchronizing oscillations to amplify epileptiform activity and drive seizure spread [4, 5]. Simultaneous electroencephalogram-functional magnetic resonance imaging (fMRI) studies confirm thalamic hyperactivation and concomitant cortical deactivation during generalized spike-wave discharges, underscoring its central role in seizure propagation [6, 7]. Moreover, thalamic dysfunction is associated with cognitive deficits in epilepsy [8]. However, the thalamic functional hierarchy in IGE remains poorly understood. Elucidating these changes may facilitate a deeper understanding of thalamic involvement in the pathophysiology of IGE.

Emerging evidence identifies the thalamus as a central hub in cerebello-cerebral communication [9]. It relays signals from the cerebellar interposed and dentate nuclei to the cerebral cortex, forming the cerebellum-related thalamo-cortical (CTC) pathway [10]. Multimodal neuroimaging studies in IGE reveal structural and functional abnormalities within the CTC pathway associated with illness duration, implicating its dysconnectivity in epileptogenesis [10, 11]. Connectivity patterns of the CTC pathway have also been linked to the therapeutic efficacy of deep-brain stimulation [2]. Research into cerebellar-thalamo-cortical interactions may elucidate the widespread effects of epileptic activity and inform therapeutic strategies in IGE.

Recent advances in connectome mapping highlight macroscopic gradients as robust markers of functional hierarchy, derived from nonlinear dimensionality reduction of functional connectivity (FC) [12, 13]. Functional gradients provide an intuitive representation of global network organization and hierarchical differentiation. Thalamo-cortical gradients reflect thalamic structural contours and are associated with behavior [14]. Gradient range changes correspond to different brain states and task demands, reflecting reconfigurations of hierarchical positioning and functional roles [15, 16]. In IGE, cerebellar-cerebral gradient shifts are associated with motor dysfunction and disease duration, suggesting that disrupted functional hierarchy may contribute to epileptic pathophysiology [14]. In contrast, eccentricity indexes regional specialization within gradient space. Altered eccentricity has been associated with default mode network-centered hierarchical reorganization, disease remission, and attentional deficits in Rolandic epilepsy [17]. While many studies report functional reorganization in epilepsy, investigations into the continuous hierarchical organization of the CTC pathway remain limited [18–20].

Currently, most functional gradient studies focus on strongly coupled connections linking core network nodes and reflecting structural architecture, supporting stable functional organization [21, 22]. However, less tightly coupled connections also carry meaningful information, contributing to network coordination, modular differentiation, integrative processes, and cognitive variability [23–25]. Methodologically, gradients based on a single connectivity range may overemphasize one coupling regime and overlook others [26]. Functionally, different connection strengths may play distinct roles in information transmission and cognitive processing [24]. Dividing FC into strength-matched bins provides a principled framework to examine hierarchical organization across coupling conditions and reduces biases from arbitrary thresholds. This approach captures gradient stability or reorganization across regimes rather than assuming a uniform hierarchy.

Despite growing interest in the functional organization of the thalamus, it remains unclear whether its hierarchy varies across connectivity strengths and is selectively disrupted in IGE. Clarifying these issues is important for understanding thalamic network dysfunction and its clinical relevance in IGE. Accordingly, this study focuses on the thalamo-cerebral and thalamo-cerebellar systems in IGE to examine whether hierarchical organization differs across connectivity strengths. Nucleus-specific alterations reflecting disrupted local integration-segregation balance were further characterized using eccentricity. This study hypothesizes that thalamic dysfunction in IGE relates to altered connectome-dependent integration-segregation, and that these alterations relate to cognitive performance.

## Materials and methods

### Participants

A total of 187 IGE patients with generalized tonic-clonic seizures were recruited from the neurology department, the Affiliated Hospital of the University of Electronic Science and Technology of China. Diagnosis was confirmed independently by two neurologists according to the International League Against Epilepsy [1]. Among them, 52 were drug-naïve, first-episode patients. All participants discontinued antiseizure medications ≥ 24 h before scanning, and none experienced seizures immediately prior. The study cohort also comprised 245 demographically matched healthy controls (HC). This study was approved by the local Ethics Committee of the University of Electronic Science and Technology of China (approval number: 2022LLYJ08, approved in 2022), and written informed consent was obtained from all participants in accordance with the Declaration of Helsinki.

### Image data acquisition

fMRI data were acquired using a 3-T scanner (Discovery MR750, GE Healthcare). Functional images were obtained using an echo-planar imaging sequence, and T1-weighted images were acquired using a 3-dimensional fast spoiled gradient echo sequence. Detailed parameters are provided in the Supplementary Material.

### Data preprocessing

Preprocessing was performed using DPABI [27]. Structural images were skull-stripped, co-registered, and segmented using DARTEL to generate deformation fields [28]. fMRI preprocessing included discarding the first five volumes, slice-timing and realignment correction, normalization to 2 mm isotropic space using DARTEL, linear detrending, nuisance regression (first five CompCor components, Friston-24 motion parameters, and global signal), scrubbing, and band-pass filtering (0.01–0.10 Hz). Further details are provided in the Supplementary Material.

### Analysis of FC profiles across connectivity strengths

Voxel-wise FC matrices were computed separately between the thalamus (number of voxels = *N*1) and cerebrum (number of voxels = *N*2), and between the thalamus and the cerebellum (number of voxels = *N*3), yielding participant-specific thalamo-cerebral (*N*1 ×* N*2) and thalamo-cerebellar (*N*1 × *N*3) matrices.

**Fig. 1:**
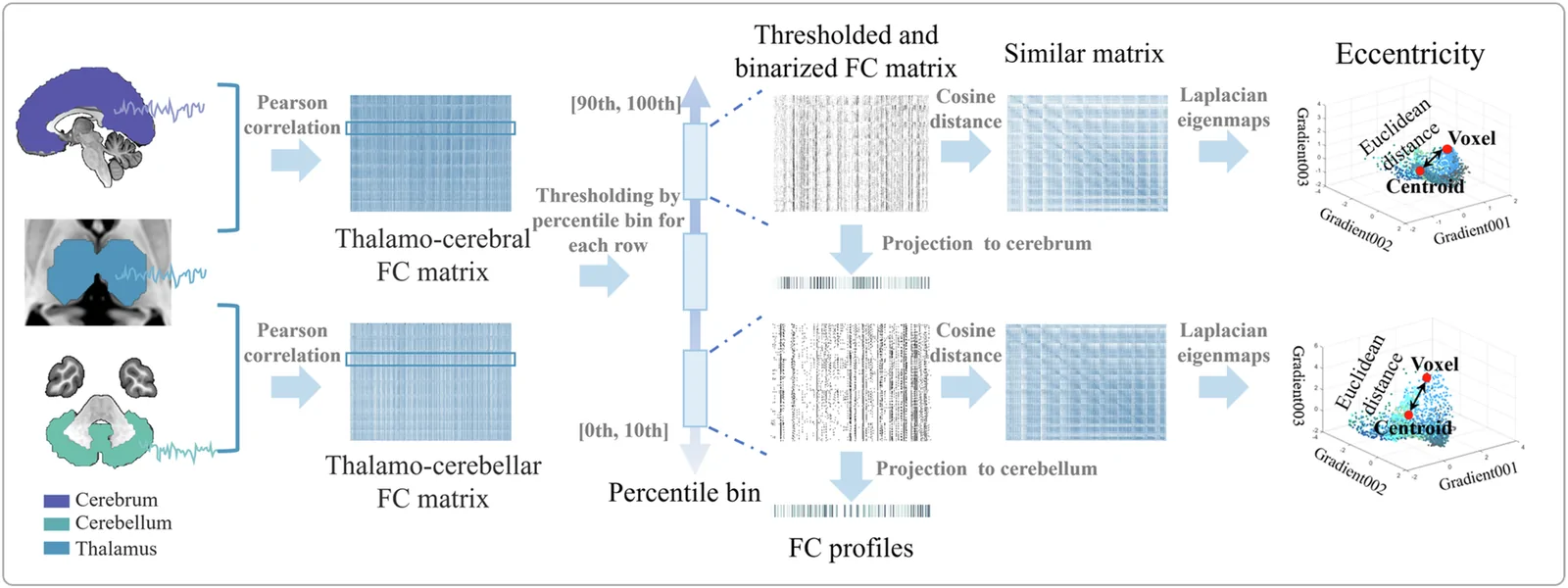
Workflow and schematic of analytical processing based on thalamo-cerebral and thalamo-cerebellar gradients and eccentricity. First, the FC of the thalamus and cerebrum, and cerebellum was calculated separately, and then the FC matrix was thresholded row by row based on the percentile range of connectivity strength (*i.e*., connectivity bin). This processing method can generate a functional connection containing specific connection subsets, which were defined according to the systematic distribution of connection strength. On this basis, the cosine distance at each voxel was calculated to construct a similarity matrix between voxels. The Laplacian eigenmaps were then used to capture the functional gradients. Subsequently, the eccentricity was calculated to characterize the hierarchical structure in the three-dimensional gradient space. Among them, the FC profile was obtained by averaging the columns of the thresholded and binarized FC matrix and projecting it onto the cerebrum or cerebellum. FC, Functional connectivity; HC, Healthy controls; IGE, Idiopathic generalized epilepsy

To capture connectivity patterns across strengths, each matrix row was divided into ten percentile-based bins (Fig. 1). Connectivity values were thresholded into percentile ranges (*e.g*., [0th, 10th] for anticorrelated and [90th, 100th] for correlated connections). Within each bin, connections were binarized by retaining edges within the corresponding range, emphasizing relative connectivity topography rather than absolute strength [25]. This procedure generated binarized thalamo-cerebral and thalamo-cerebellar matrices for each subject.

FC profiles of the cerebrum (1 × *N2*) and cerebellum (1 × *N3*) were derived by column-wise averaging of binarized matrices, integrating thalamic connectivity for each cerebral and cerebellar region. To examine the network composition of these profiles, the top 10% highest-connected voxels were defined as core regions and mapped to functional networks using Yeo’s cortical parcellation [29] and Buckner’s cerebellar atlas [30]. To account for network size, the proportion of core voxels in each network was normalized by network voxel counts to obtain a relative enrichment proportion. Group differences between the IGE and HC groups were assessed using the Mann–Whitney *U* test with Bonferroni correction (*p* < 0.05).

### Functional gradient analyses

FC gradients were computed using the BrainSpace toolbox in MATLAB [31]. Cosine similarity was first computed from binarized connectivity matrices to estimate pairwise voxel-wise connectivity profile similarity. Laplacian eigenmaps were then applied to derive thalamo-cerebral and thalamo-cerebellar gradients. Furthermore, a group-level gradient template was constructed from the averaged FC matrix, and individual gradients were aligned to this template using Procrustes rotation to enable group-level comparisons. Gradients were z-scored for standardization. Group differences in gradient distributions between IGE and HC were assessed at each bin using Kolmogorov–Smirnov tests with Bonferroni correction (*p* < 0.05).

### Eccentricity analysis

For each voxel, eccentricity was defined as the Euclidean distance from its position to the centroid in the three-dimensional gradient space formed by the first three gradients. Voxel-wise eccentricity maps were spatially smoothed before group-level analysis. Group differences between IGE and HC were assessed for each connectivity bin using a general linear model, with age, gender, and mean frame-wise displacement included as nuisance regressors [32]. The mean frame-wise displacement was quantified following Power et al [33] (see Supplementary Material). Multiple comparisons were corrected using the false discovery rate, with a significance level of 0.05 after correction.

### Correlations between eccentricity and clinical variables and working memory performance

In IGE patients, associations between eccentricity values showing significant between-group differences and clinical variables (age at onset and disease duration) were examined using partial correlation, controlling for sex, age, and head motion.

Working memory performance (reaction time and accuracy) during the *n*-back task was assessed as described by Qin et al [34]. Associations between eccentricity and task performance were examined using the same covariates. Correlation significance was set at Bonferroni-corrected *p* < 0.05.

### Sensitivity and robustness analyses

To assess the robustness, analyses were repeated using weighted instead of binarized connectivity matrices.

Given the ongoing debate regarding global signal regression (GSR) and its variable use in functional gradient studies [14, 35], analyses were also repeated without GSR. Consistency between results obtained with and without GSR was evaluated using Spearman correlation.

## Results

### Demographic information and clinical characteristics

Due to excessive head motion and poor data quality (*e.g*., incomplete cerebellar coverage, blurred T1 images, or poor registration), 23 HC and 43 IGE participants were excluded. The final sample included 222 HC and 144 IGE patients. No significant group differences were observed in age or sex. Detailed demographic and clinical characteristics are provided in Table 1.

**Table 1:** Demographic characteristics of idiopathic generalized epilepsy (IGE) patients and healthy controls (HC)

Characteristic	IGE	HC	*p*-value
	Mean (SD)	Mean (SD)	
Number	144	222	-
Age (years)	25.87 ± 11.69	26.03 ± 6.56	0.868^a^
Sex (male:female)	64:80	111:111	0.299^b^
ASMs (with:without)	92:52	-	-
Age at onset (years)	20.26 ± 11.84	-	-
Illness duration (years)	5.40 ± 8.19	-	-
mFD (mm)	0.12 ± 0.05	0.09 ± 0.04	-

*ASMs* Antiseizure medications, *mFD* Mean frame-wise displacement, *SD* Standard deviation

^a^ The *p*-value was obtained by a two-sample *t*-test

^b^ The *p*-value was obtained by a χ^2^ test

### Aberrant FC profiles across connectivity strengths in IGE patients

**Fig. 2:**
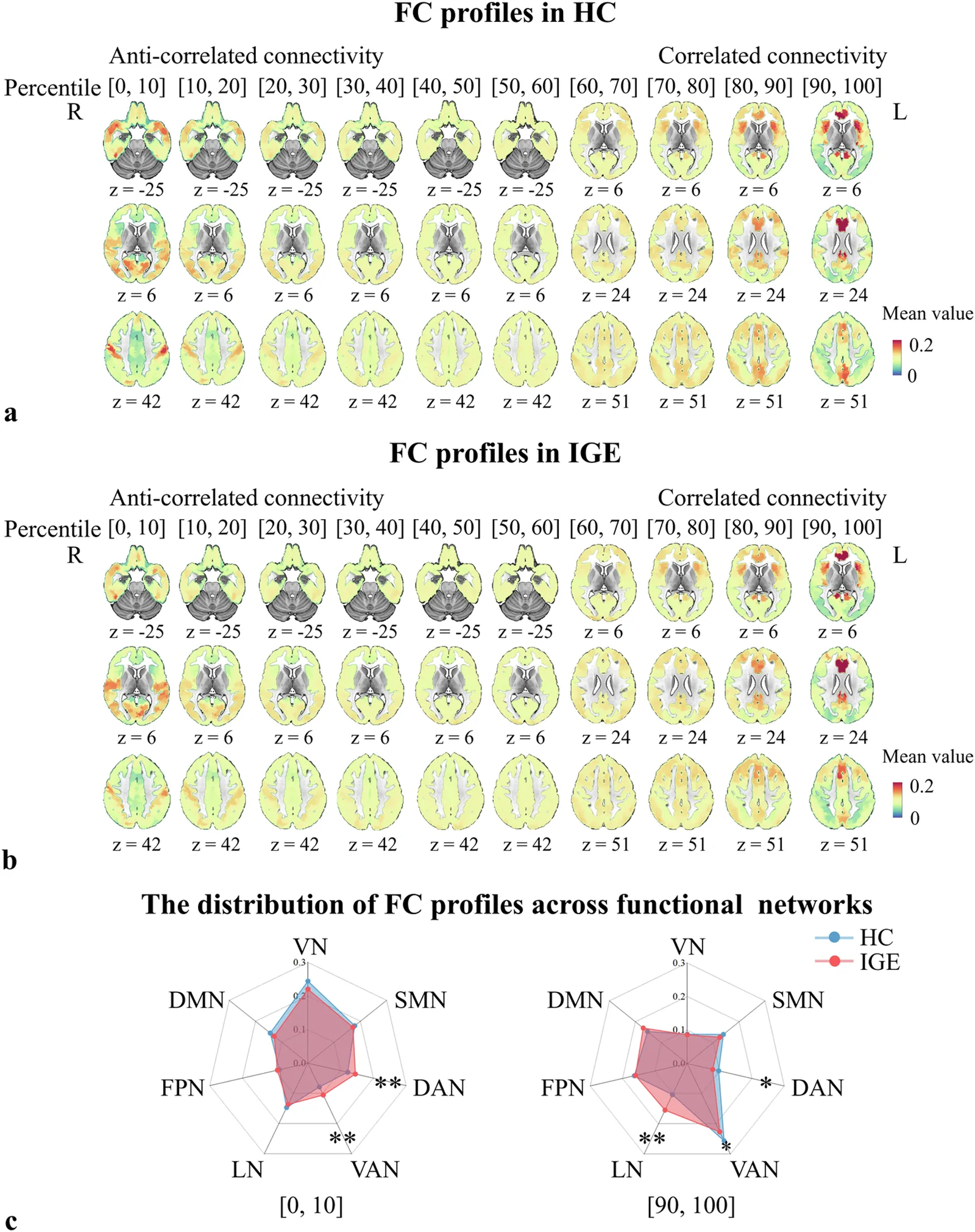
**a** Thalamo-cerebral FC profiles of HC at all connectivity strengths were obtained by averaging the thresholded and binarized FC matrix. **b** Thalamo-cerebral FC profiles of IGE under all connectivity strengths. **c** The network distribution of the top 10% strongest connected voxels in the FC profile. Group differences were assessed using the Mann–Whitney *U* test. * Uncorrected *p* < 0.05; ** Bonferroni-corrected *p* < 0.05; red represents IGE, and blue represents HC. DAN, Dorsal attention network; DMN, Default mode network; FC, Functional connectivity; FPN, Frontoparietal network; HC, Healthy controls; IGE, Idiopathic generalized epilepsy; L, Left; LN, Limbic network; R, Right; SMN, Sensorimotor network; VAN, Ventral attention network; VN, Visual network

Thalamo-cerebral FC profiles in both groups were broadly distributed across networks under intermediate bins, but distinct dominant patterns emerged under anticorrelated and correlated bins (Fig. 2a, b). Specifically, anticorrelated connections targeted primary sensorimotor, visual, and orbitofrontal regions, whereas correlated connections involved the supplementary motor area, cingulate cortex, insula, and middle frontal gyrus.

Network-level analysis of the top 10% strongest connections showed that, under the [0, 10] bin, IGE patients exhibited higher proportions within the dorsal and ventral attention network (DAN, VAN) than HC, whereas under the [90, 100] bin, VAN and DAN proportions were reduced and limbic network proportion increased (Fig. 2c). Across intermediate bins, IGE showed increased VAN and decreased limbic network ([10, 20] to [40, 50]) and increased default mode and visual networks with lower VAN and DAN ([50, 60] to [80, 90]) (Supplementary Fig. S2 and Table S1).

**Fig. 3:**
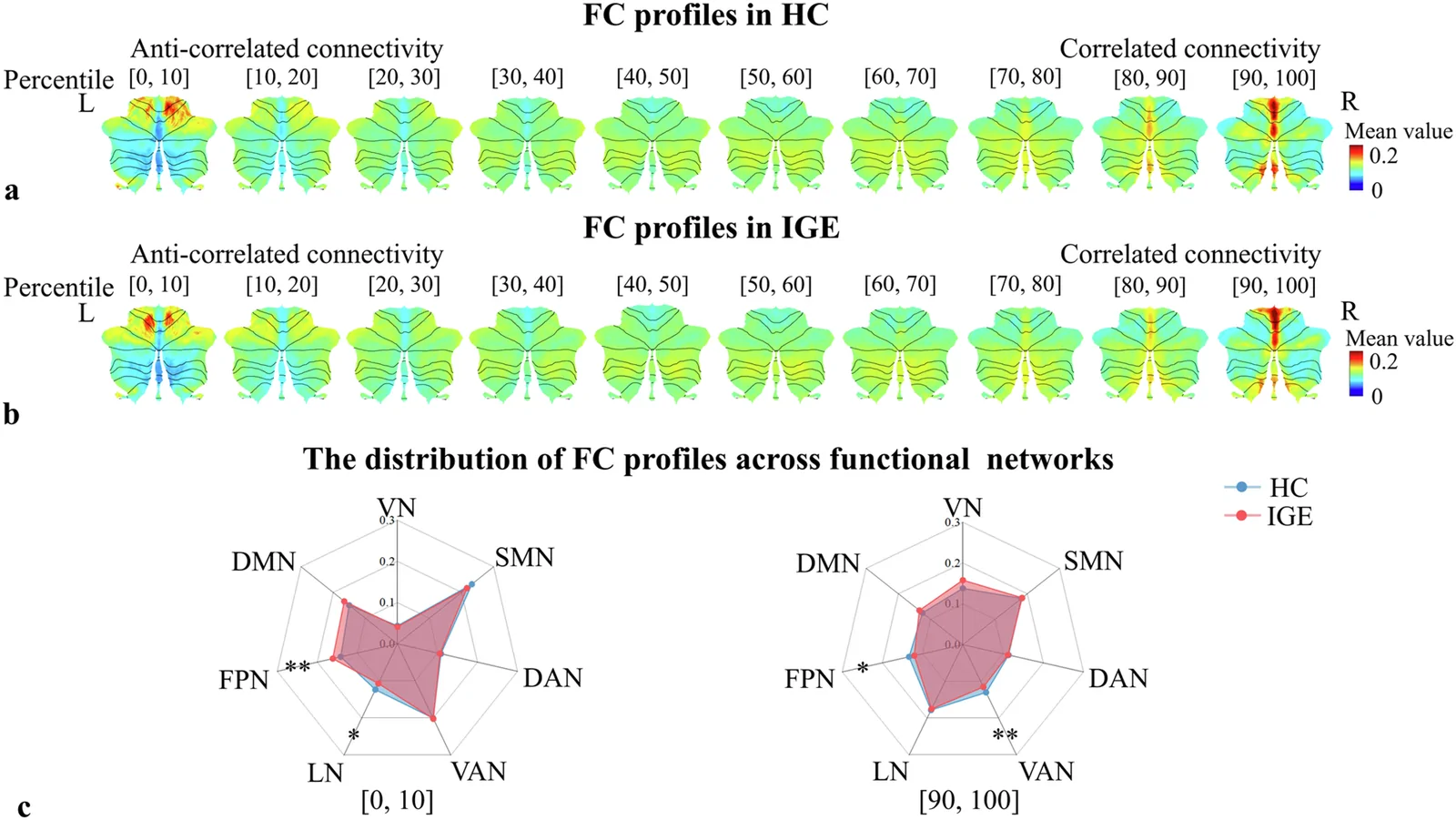
**a** Thalamo-cerebellar FC profiles of HC under varying connectivity strengths were obtained by averaging the thresholded and binarized FC matrix. **b** Thalamo-cerebellar FC profiles of IGE under varying connectivity strengths. **c** The network distribution of the top 10% strongest connected voxels in FC profile. Group differences were assessed using the Mann–Whitney *U* test. * Uncorrected *p* < 0.05; ** Bonferroni-corrected *p* < 0.05; red represents IGE, and blue represents HC. DAN, Dorsal attention network; DMN, Default mode network; FC, Functional connectivity; FPN, Frontoparietal network; HC, Healthy controls; IGE, Idiopathic generalized epilepsy; L, Left; LN, Limbic network; R, Right; SMN, Sensorimotor network; VAN, Ventral attention network; VN, Visual network

Similarly, thalamo-cerebellar FC profiles exhibited uniform distributions across cerebellar regions under intermediate bins. Low-percentile connections preferentially involved lobules I-IV, V, IX, and the lateral Crus I, whereas high-percentile connections shifted toward the posterior part of Crus I, lobules VIIIb–X, and vermis (Fig. 3a, b).

Compared to HC, IGE exhibited altered cerebellar network distributions across bins. Specifically, for the [0, 10] bin, IGE patients showed increased frontoparietal network and decreased limbic network proportions. In contrast, for the [90, 100] bin, frontoparietal network and VAN proportions were reduced (Fig. 3c). In the other bins, reduced VAN, frontoparietal, and visual proportions and increased default mode and limbic networks were observed (Supplementary Fig. S3 and Table S2).

### Disrupted thalamic functional gradient in IGE patients

**Fig. 4:**
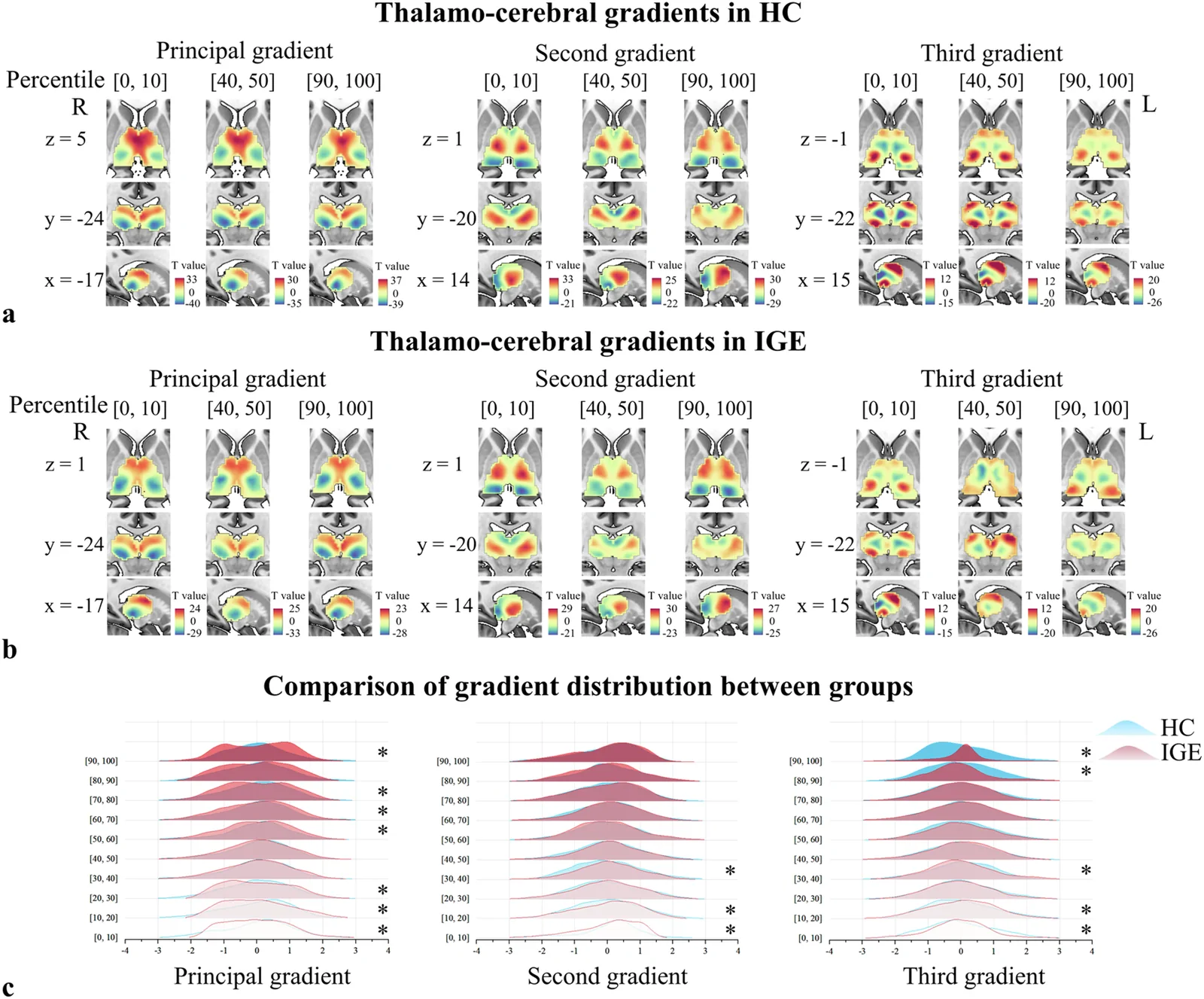
**a** Thalamo-cerebral gradients in HC. **b** Thalamo-cerebral gradients in IGE. Only gradients for anticorrelated, intermediate, and correlated connectivity strengths were shown. **c** The gradient distribution between groups was compared under varying connectivity strengths using the Kolmogorov–Smirnov test. * Bonferroni-corrected *p* < 0.05; red represents the gradient distribution of IGE, and blue represents the gradient distribution of HC. HC, Healthy controls; IGE, Idiopathic generalized epilepsy; L, Left; R, Right

The first three gradients, explaining over 75% of the total variance (Supplementary Figs. S4 and S5), were analyzed. In thalamo-cerebral gradients, the principal gradient in IGE followed a dorsomedial/ventrolateral axis under anticorrelated and correlated conditions but shifted to a dorsoanterior/ventroposterior axis at intermediate range (Fig. 4a, b). The second gradient aligned along a medial/lateral axis under [10, 20] and [50, 60] bins, and along an anterior/posterior axis under other bins. The third gradient generally followed the principal gradient axis, but its dorsomedial portion diverged toward dorsoanterior and ventroposterior regions, forming a bilaminar polarity-inverted layered structure (Supplementary Table S3).

**Fig. 5:**
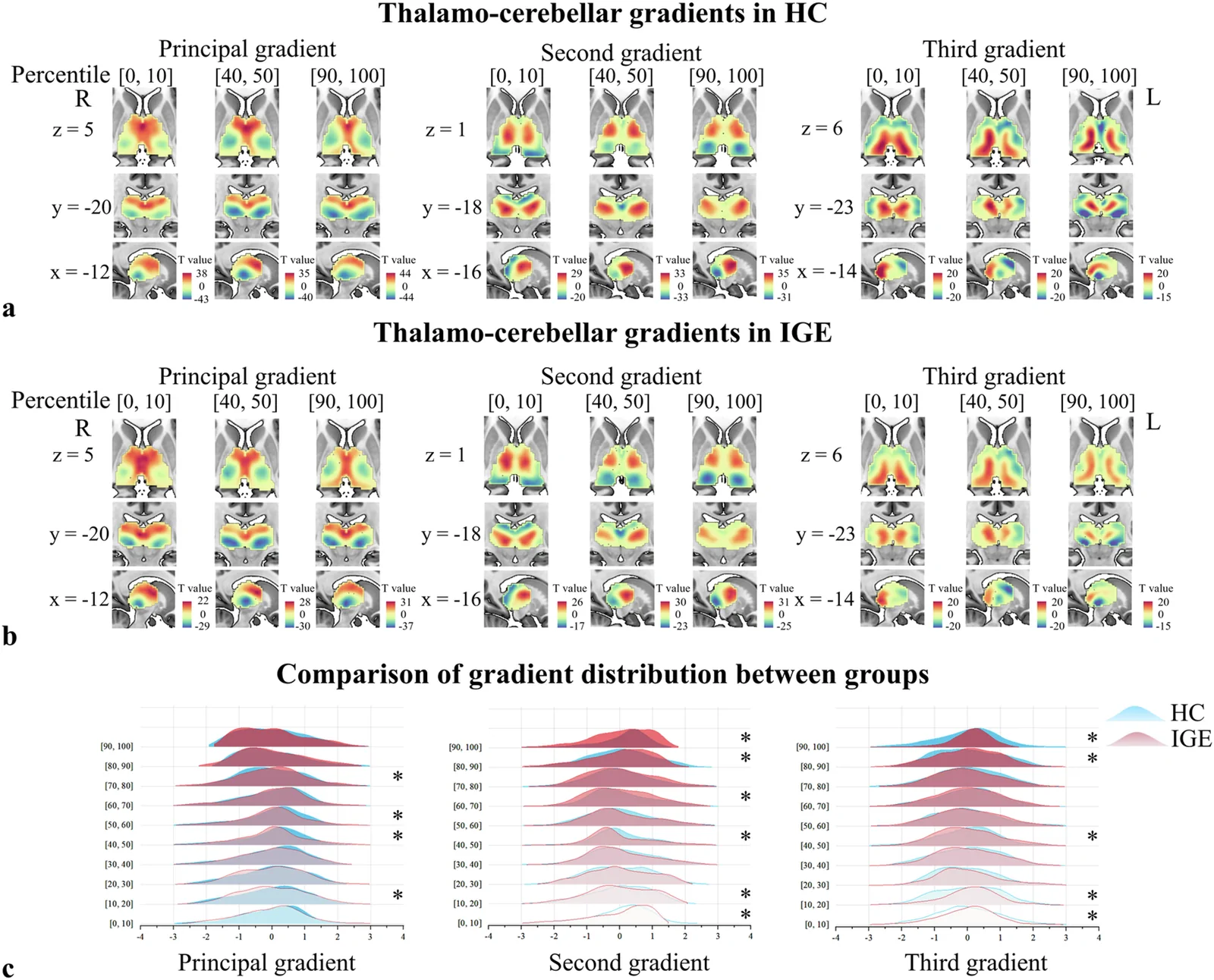
**a** Thalamo-cerebellar gradients in HC. **b** Thalamo-cerebellar gradients in IGE. Only gradients for anticorrelated, intermediate, and correlated connectivity strengths were shown. **c** The gradient distribution between groups was compared under varying connectivity strengths using the Kolmogorov–Smirnov test. * Bonferroni-corrected *p* < 0.05; red represents the gradient distribution of IGE, and blue represents the gradient distribution of HC. HC, Healthy controls; IGE, Idiopathic generalized epilepsy; L, Left; R, Right

Thalamo-cerebellar gradients showed comparable organizations for the first two gradients, whereas the third gradient differed across connectivity ranges (Fig. 5a, b). Under the [0, 10] bin, it aligned along the anterolateral/posteromedial axis, whereas under the [90, 100] connectivity bin, its orientation resembled the principal gradient (dorsomedial/ventrolateral axis) and displayed a bilaminar polarity-inverted layered structure, with the ventrolateral region diverging toward dorsomedial and ventrolateral areas (Supplementary Table S4).

Group differences were observed under anticorrelated conditions across all gradients, whereas under correlated conditions, group differences were absent in the thalamo-cerebral second gradient and the thalamo-cerebellar principal gradient (Figs. 4c and 5c).

### Altered thalamic eccentricity in IGE patients

**Fig. 6:**
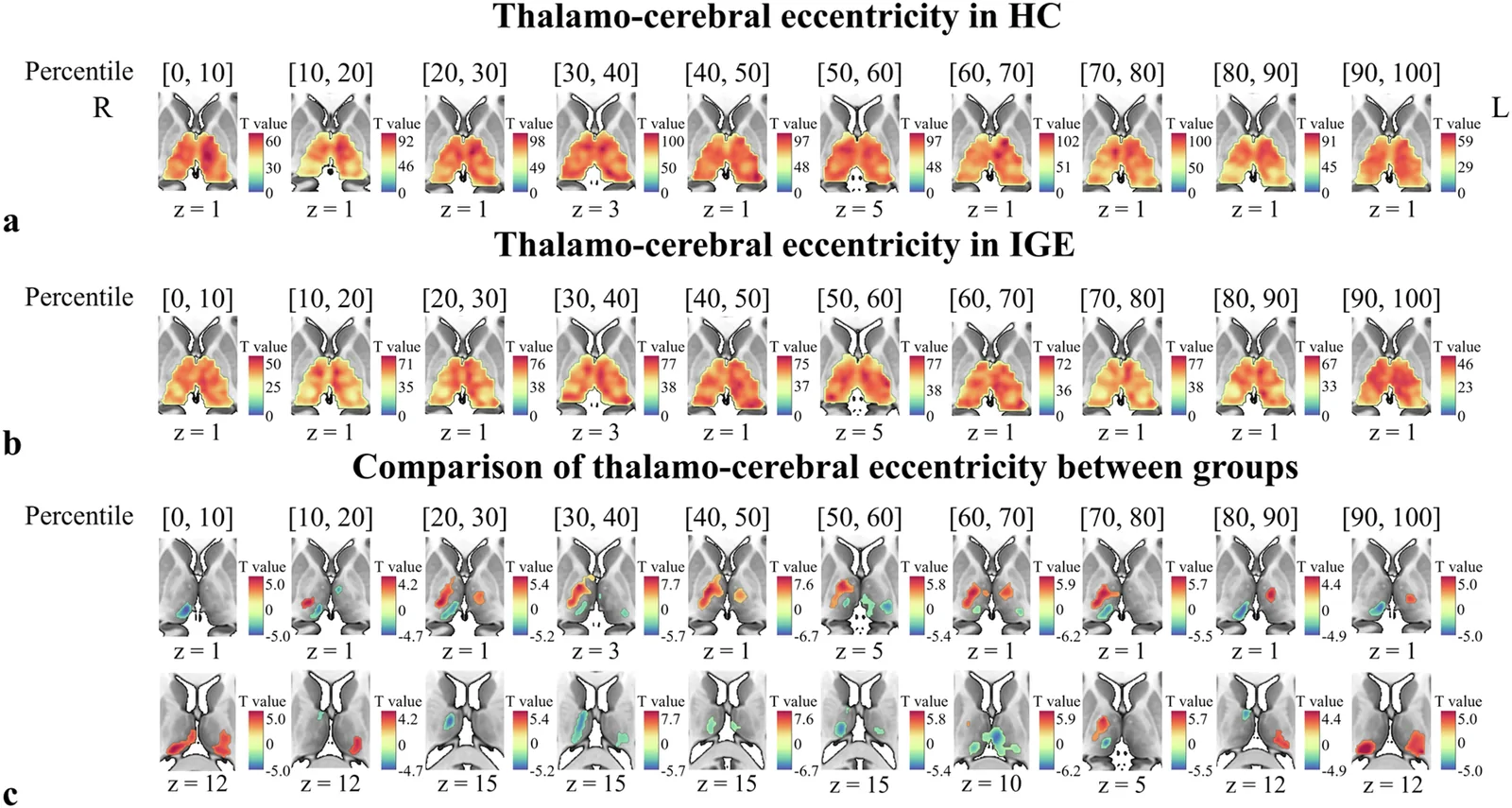
**a** Eccentricity of the thalamo-cerebral gradient space in HC under varying connectivity strengths. **b** Eccentricity of the thalamo-cerebral gradient space in IGE under varying connectivity strengths. **c** The difference in eccentricity between IGE and HC (FDR-corrected *p* < 0.05). FDR, False discovery rate; HC, Healthy controls; IGE, Idiopathic generalized epilepsy; L, Left; R, Right

**Fig. 7:**
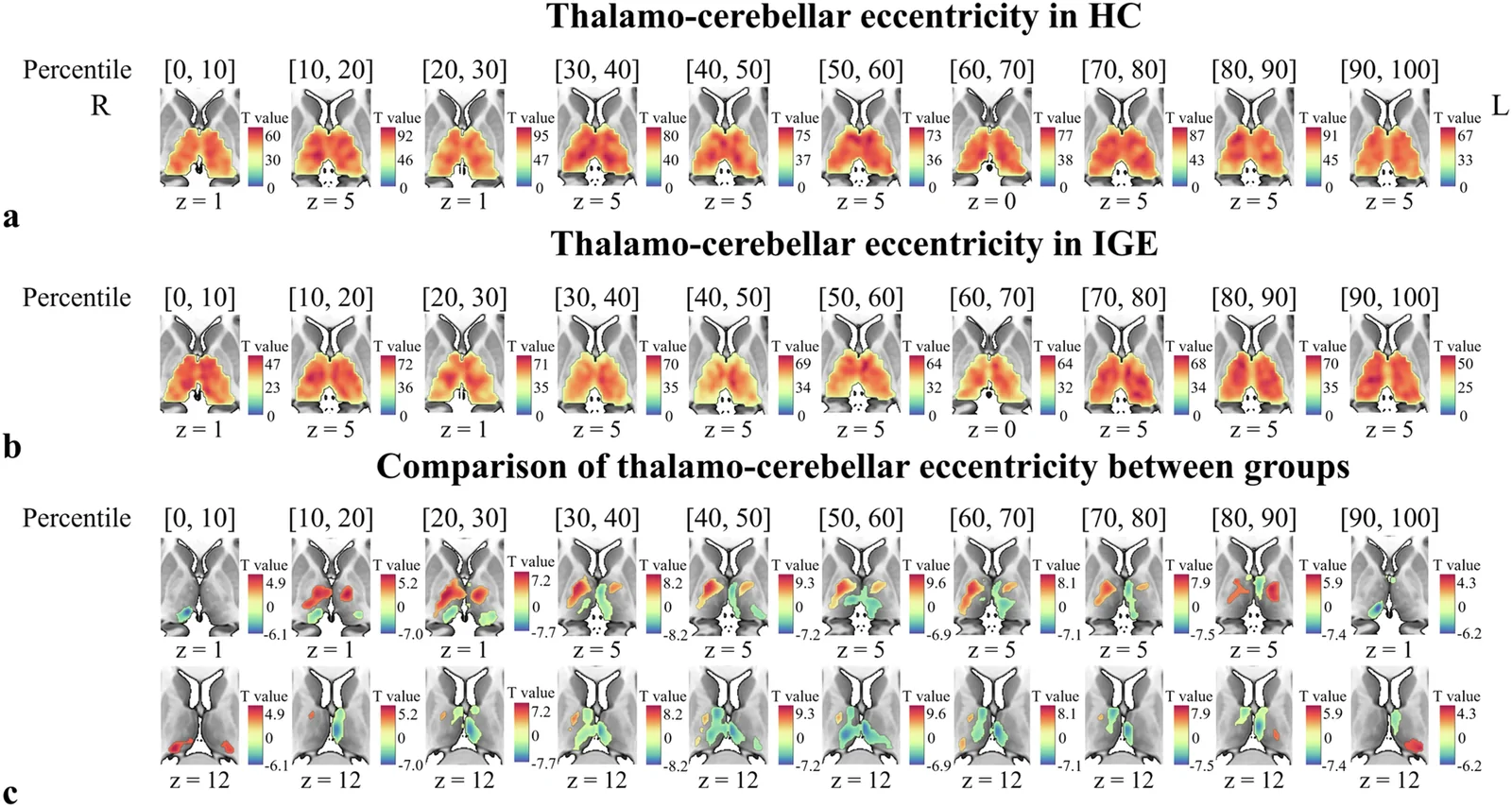
**a** Eccentricity of the thalamo-cerebellar gradient space in HC under varying connectivity strengths. **b** Eccentricity of thalamo-cerebellar gradient space in IGE under varying connectivity strengths. **c** The difference in eccentricity between IGE and HC (FDR-corrected *p* < 0.05). FDR, False discovery rate; HC, Healthy controls; IGE, Idiopathic generalized epilepsy; L, Left; R, Right

Thalamo-cerebral and thalamo-cerebellar eccentricity across connectivity strengths were shown in Figs. 6 and 7. In IGE, a shared cluster of decreased eccentricity relative to HC was identified under anticorrelated and correlated conditions, primarily in the right center median nucleus (CM) and medial pulvinar (PuM). Increased eccentricity emerged in both pathways under the [0, 10] and [90, 100] bins, with a shared cluster predominantly located in the left lateral posterior nucleus (LP) and PuM. Additional right-lateralized clusters were detected in thalamo-cerebral eccentricity under [0, 10] and [90, 100] bins, as well as in thalamo-cerebellar eccentricity under the [0, 10] bin. Other bins also showed increases, primarily involving the ventral anterior nucleus parvocellular part, ventral lateral anterior nucleus, ventral lateral ventral part, ventral medial nucleus, and ventral posterior medial nucleus. Nuclear composition was defined using the Morel atlas [36] (see the Supplementary Material).

Notably, a specific decrease in thalamo-cerebellar eccentricity was observed under the [90, 100] bin, with the cluster primarily located in the left mediodorsal nucleus parvocellular part (MDpc), the central lateral nucleus (CL), and the anteroventral nucleus (AV) (Supplementary Tables S5 and S6). This cluster was absent in the thalamo-cerebellar [0, 10] bin and in the thalamo-cerebral [0, 10] and [90, 100] bins.

### Correlation with clinical variables and cognitive performance

**Fig. 8:**
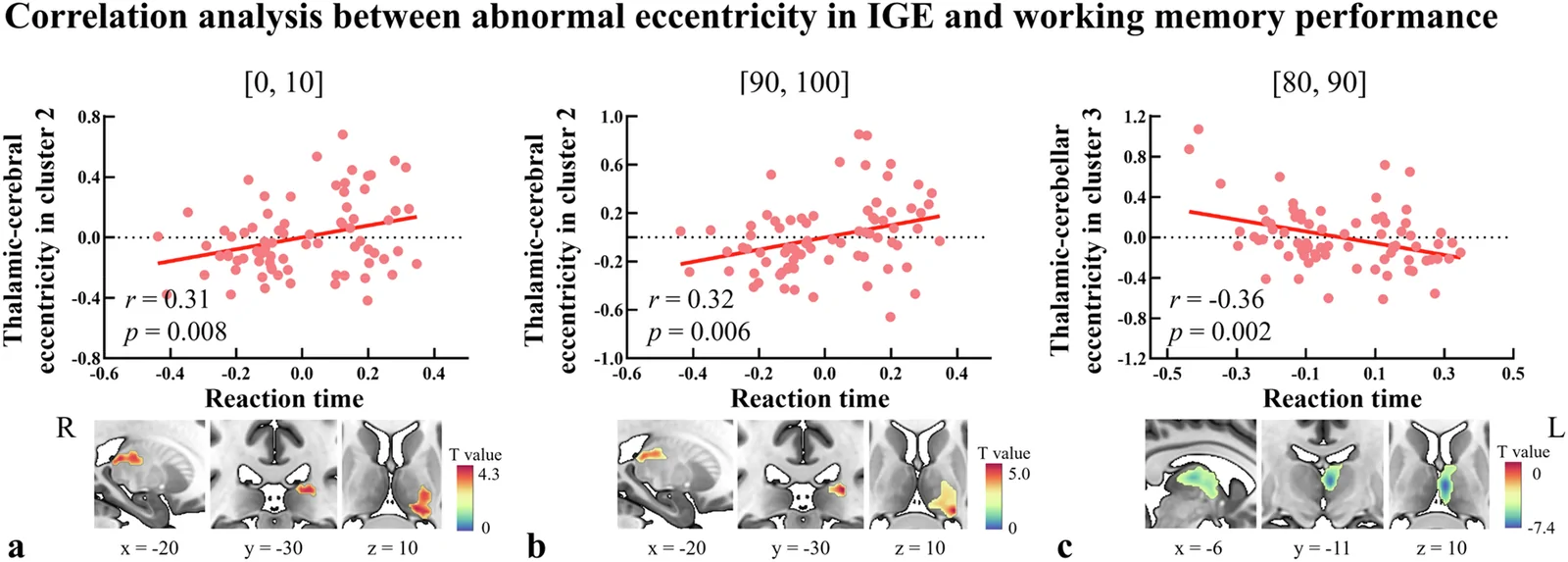
Correlation analysis between abnormal eccentricity in IGE and working memory performance across connectivity strengths (Bonferroni correction, *p* < 0.05). **a** Under the [0, 10] connectivity bin, reaction time was positively correlated with thalamo-cerebral eccentricity in cluster 2, primarily located in the left PuM, LP, and VPLp. **b** Under the [90, 100] connectivity bin, a similar positive correlation was observed for cluster 2, located in a region spatially consistent with that in the [0, 10] connectivity bin. **c** Under the [80,90] connectivity bin, reaction time was negatively correlated with thalamo-cerebellar eccentricity in cluster 3, mainly involving the left AV, CL, MDpc, VApc, and VLpd. Partial correlation analysis was performed, controlling for age, sex, and head motion. Residuals are plotted for visualization. AV, Anterior ventral nucleus; CL, Central lateral nucleus; LP, Lateral posterior nucleus; MDpc, Mediodorsal nucleus parvocellular part; PuM, Medial pulvinar nucleus; VApc, Ventral anterior nucleus parvocellular part; VLpd, Ventral lateral dorsal part; VPLp, Lateral geniculate nucleus posterior part

No significant correlations were found between eccentricity and clinical variables after Bonferroni correction. Interestingly, two thalamo-cerebral clusters showing significant intergroup differences were positively associated with reaction time, including cluster 2 under the [0, 10] bin (Fig. 8a) and cluster 2 under the [90, 100] bin (Fig. 8b), both primarily located in the left LP, PuM, and lateral geniculate nucleus posterior part. Additionally, under the [80, 90] bin, decreased eccentricity in thalamo-cerebellar cluster 3 was negatively correlated with reaction time (Fig. 8c), primarily located in the left AV, CL, MDpc, ventral anterior nucleus parvocellular part, and ventral lateral dorsal part.

### Sensitivity and robustness analysis

Binarization exerted minimal impact on thalamo-cerebral and thalamo-cerebellar FC profiles, with deviations limited to the [50, 60] and [40, 50] bins corresponding to near-zero FC values (Supplementary Figs. S6 and S8). Between-group differences in network composition derived from non-binarized FC were consistent with binarized results (Supplementary Figs. S9 and S10). Functional gradients and between-group differences were largely preserved, with instabilities limited to the thalamo-cerebellar third gradient and several intermediate bins (Supplementary Figs. S11 and S12). Eccentricity results were also robust, with discrepancies mainly confined to intermediate bins (Supplementary Figs. S13 and S14).

FC profiles were highly consistent across preprocessing strategies with and without GSR (*r* > 0.36, *p* < 0.001; Supplementary Fig. S15). In contrast, gradients showed variable sensitivity to GSR across systems, bins, and gradient orders (see Supplementary Material for results). The principal and second gradients were generally stable in both systems (*r* > 0.40), whereas the third gradient showed reduced stability in specific intermediate or anticorrelated bins (Supplementary Fig. S16). GSR also modulated group differences in gradient distributions, with partial disappearance or emergence of effects across bins, particularly in intermediate ranges (Supplementary Fig. S17). Further detailed descriptions are available in the Supplementary Results. Notably, eccentricity remained highly robust, showing strong correlations across preprocessing strategies (*r* > 0.6) and largely consistent between-group differences (Supplementary Figs. S18 and S19).

## Discussion

Connectome gradient and eccentricity analyses revealed abnormal organization of thalamo-cerebral and thalamo-cerebellar networks across connectivity strengths in IGE. Specifically, disrupted connectome-dependent reorganization of FC profiles and functional hierarchy indicates an altered integration-segregation balance in IGE. Region-specific eccentricity abnormalities further reflected disrupted integration and segregation in thalamic nuclei associated with working memory deficits. Notably, partially overlapping yet pathway-specific alterations in thalamo-cerebral and thalamo-cerebellar pathways across connectivity strengths may provide a systems-level substrate for the motor and cognitive deficits in IGE. Together, these findings highlight disrupted thalamic functional hierarchy in IGE and its mechanistic link to disease-related dysfunction.

### Connectome-dependent reorganization of FC Profiles

In HC, the thalamus exhibited connectome-dependent reorganization of FC profiles across connectivity strengths. Under anticorrelated connections, thalamic interactions mainly involved sensorimotor and limbic networks, reflecting the role of first-order nuclei in sensory integration [37, 38]. In contrast, correlated connections preferentially engaged higher-order cognitive networks, consistent with top-down modulation by mediodorsal and pulvinar nuclei [9]. At intermediate strengths, FC profiles were more evenly distributed, suggesting a non-selective coupling state. These patterns highlight the thalamus as an integrative hub enabling flexible coordination between sensory and cognitive processing [9, 39], balancing segregation and integration for hierarchical information transfer [40, 41]. Non-binarized analyses yielded largely consistent results. Non-binarized analyses showed largely consistent spatial patterns, with apparent negative correlations in anticorrelated bins reflecting sign inversion rather than spatial inconsistency. Despite differences in sign handling, key regions showed high spatial overlap across approaches.

Disruption of the integration-segregation balance may be associated with motor and cognitive impairments in epilepsy [14, 42]. In this study, IGE showed connectome-dependent thalamo-cortical abnormalities. Under anticorrelated conditions, thalamo-attentional coupling increased, and thalamo-limbic coupling decreased, with the opposite pattern under correlated conditions, suggesting a failure of the thalamus to flexibly regulate interactions across functional systems. Previous research reported reduced dynamic regulation of thalamo-cortical coupling in IGE, potentially reflecting altered oscillatory mechanisms arising from interactions between excitatory relay and inhibitory thalamic reticular neurons [43, 44]. Impaired regulation may limit the thalamic ability to modulate segregation and integration, resulting in connectome-dependent abnormal reorganization. Similar abnormalities in thalamo-cerebellar pathways indicated disrupted coordination within cognitive-motor circuits. Overall, thalamic dysfunction in IGE reflects a failure of cross-state regulatory control rather than a single-network abnormality, potentially linking to its multidimensional clinical manifestations.

### Thalamic functional hierarchy impairment in IGE

Abnormal generalized spike-wave discharges in IGE propagate rapidly across distributed networks, altering large-scale connectome organization [19]. Connectome gradient analysis provides an integrative framework for characterizing functional hierarchy alterations in relation to cognitive and behavioral abnormalities [45, 46]. Here, patients showed aberrant thalamo-cerebral and thalamo-cerebellar gradient distributions across connectivity strengths.

Under correlated conditions, the principal thalamo-cerebral gradient and second thalamo-cerebellar gradient were expanded, whereas the third gradients of both systems were compressed. This pattern was robust across preprocessing strategies, suggesting a non-uniform reorganization of thalamic functional hierarchies across different functional axes. The expansion of the first two gradients reflects stronger differentiation between thalamic subsystems, indicating increased segregation and reduced cross-hierarchical integration. Previous research has shown that the principal gradient is closely associated with thalamic anatomy and gray matter volume [14], and gray matter volume reduction and microstructural abnormalities in the thalamus have been consistently reported in IGE patients [47, 48]. Thus, the abnormal pattern of functional gradients may have a structural basis, and future studies could further investigate the direct relationship between thalamic atrophy and gradient alterations. Moreover, the antero-posterior axis of the thalamic gradient primarily aligns with the higher-order association-sensorimotor axis [14, 49, 50]. Increased separation along this axis may impair information integration between higher-order cognitive and lower-order perceptual regions, leading to cross-network coordination deficits. This may provide a network-level explanation for the executive dysfunction and sensorimotor deficits commonly observed in IGE patients. Furthermore, the compression of the third gradients suggests increased integration of ventrolateral and anteroventral/ventroposterior thalamic regions. The thalamus plays a central role in coordinating the cerebello-thalamo-cortical pathway, which modulates cortical excitability and large-scale synchronization [10, 51]. In this context, the observed increase in integration may represent a maladaptive reorganization that facilitates rapid cross-system interactions, thereby promoting the widespread synchronization of spike-wave discharges in IGE. Although this hypothesis requires further validation, it offers new theoretical insights into the pathophysiological mechanisms of IGE.

Additionally, connectome-dependent gradient alterations were observed in IGE. Because functional gradients are highly stable across connectivity strengths in HC [25], the bin-specific intergroup differences across bins likely reflect epilepsy-related disruptions in thalamic hierarchical regulation under different coupling modes. Without binarization, reduced stability in intermediate bins may reflect greater noise vulnerability of near-zero correlations. In this context, topology-based connectivity ranking mitigates this instability, thereby providing a more robust representation of thalamic organization. Notably, between-group differences were sensitive to GSR, especially under intermediate strengths. Nonetheless, IGE patients still exhibited connectome-dependent alterations in gradient distributions, reflecting an abnormal dependence of gradient patterns on connectivity strength. These findings suggest impaired cross-network coordination and altered multi-level thalamic regulation in IGE.

### Abnormalities of specific nuclei in the thalamus in IGE

Eccentricity alterations provide localized evidence of disrupted integration-segregation within the thalamus in IGE. Decreased eccentricity indicates concentrated organization and reduced segregation, and increased eccentricity suggests enhanced differentiation with reduced cross-level integration and has been associated with decreased information-processing efficiency [52]. Here, CM, PuM, and LP showed consistent abnormalities across connectivity strengths and pathways, indicating widespread involvement of the CTC circuit [53]. Specifically, decreased eccentricity in the CM suggests diminished segregation. Previous work has shown that the CM has extensive direct connections with the striatum and somatosensory cortex [54], and decreased segregation may impair the coordination efficiency of the striato-thalamo-cortical sensorimotor loop in IGE. In contrast, increased eccentricity in the LP indicates enhanced segregation within the functional hierarchy. Given the role of LP in sensory integration, spatial attention, and seizure propagation [55–58], this abnormality may impair sensory-attentional integration and facilitate aberrant network synchronization in IGE. Collectively, eccentricity alterations across different thalamic nuclei converge on a disruption of the integration-segregation balance within the thalamus in IGE. This hierarchical imbalance may be associated with impaired attentional control in IGE and may increase seizure susceptibility by promoting large-scale network synchronization. These findings were robust across different preprocessing strategies, reinforcing the reliability of eccentricity as a reproducible and quantifiable metric. Notably, thalamo-cerebral eccentricity correlated with prolonged working memory reaction times, highlighting its sensitivity to hierarchical network alterations and cognitive outcomes in IGE.

However, the intergroup differences in certain thalamic nuclei were not fully consistent across connectivity conditions. This variability may reflect distinct functional roles of different connectivity strengths and involvement in multi-level pathological processes. It has been reported that gradient patterns under intermediate connectivity conditions have been shown to better capture individual variability [25]. Future studies could further distinguish consistently affected nuclei from condition-specific ones and examine their associations with cognitive function, individual variability, and multimodal features to further elucidate the implications of functional hierarchy impairment in IGE.

Under non-anticorrelated conditions, a cluster of decreased eccentricity was identified in the MDpc, CL, and AV within the thalamo-cerebellar pathway, highlighting a potential role of the cerebellum in functional network reorganization in IGE. The cerebellum controls motor coordination, and the CL is one of its primary thalamic targets. Disruption of the cerebello-thalamic tracts projecting to the CL impairs motor coordination and grasping ability [59]. Thus, decreased eccentricity in the CL may reduce thalamic-cerebellar information integration, potentially relating to motor dysfunction associated with recurrent seizures. Moreover, the CL is involved in arousal regulation [56, 60], and its dysfunction may be linked to transient consciousness impairment in IGE. Meanwhile, the AV and MDpc participate in cognitive, emotional, and executive processes [61–63] and have also been implicated in seizure regulation [64, 65]. In this study, decreased eccentricity in these nuclei suggests impaired segregation, which may affect emotional-cognitive regulation and may reduce their capacity to modulate epilepsy-related abnormal network activity. Supporting this, thalamo-cerebellar eccentricity was negatively associated with reaction time under correlated conditions, linking impaired integration to cognitive inefficiency. These findings suggest that disruption of the cerebello-thalamic loop may underlie slower information processing and working memory deficits in IGE, providing a system-level explanation for the cognitive inefficiency commonly observed in these patients.

### Limitations

This study has several limitations. First, the cross-sectional design precludes determining whether the gradient alterations are a cause, consequence, or compensation of epilepsy. Second, the relatively low spatial resolution of the fMRI data may limit the spatial precision and reliability of the results. Third, working memory performance was not assessed in the HC of this cohort. Comparison with an independent age- and sex-matched HC sample showed significantly lower accuracy and longer reaction times in IGE (Supplementary Fig. S20). Nevertheless, the lack of broader cognitive and motor assessments limits direct interpretation of brain-behavior relationships. Finally, antiseizure medication effects could potentially influence the results. Additional analyses controlling for medication status yielded largely consistent findings (Supplementary Fig. S21), but variability in dosage and treatment duration could not be fully addressed.

## Conclusions

In conclusion, this study systematically examined the thalamic functional hierarchy across multiple connectomes in IGE. Connectome-dependent alterations in FC profiles and widespread thalamic gradient abnormalities reflected disrupted integration-segregation balance. Eccentricity analysis revealed that region-specific thalamic deviations were associated with working memory performance, providing a potential neural substrate for cognitive dysfunction in IGE. Crucially, convergent thalamo-cerebellar and thalamo-cerebral eccentricity alterations implicate the CTC circuit in IGE pathophysiology. Collectively, these findings advance understanding of thalamic functional hierarchy disruptions and provide a framework for investigating integration-segregation imbalances in epileptic networks.

## Supplementary information

**Additional File 1: Fig. S1.** Number of timepoints remaining after preprocessing for each participant. Notes: red represents IGE, and blue represents HC. **Fig. S2.** Distribution of thalamo-cerebral FC profiles across networks and group comparisons under varying connectivity strengths. Notes: * indicates uncorrected *p* < 0.05, ** indicates Bonferroni-corrected *p* < 0.05; red represents IGE, and blue represents HC. **Fig. S3.** Distribution of thalamo-cerebellar FC profiles across networks and group comparisons under varying connectivity strengths. Notes: * indicates uncorrected *p* < 0.05, ** indicates Bonferroni-corrected *p* < 0.05; red represents IGE, and blue represents HC. **Fig. S4.** Variance explained by thalamo-cerebral gradients across a full range of connectivity strengths. Notes: red represents IGE, and blue represents HC. **Fig. S5.** Variance explained by thalamo-cerebellar gradients across a full range of connectivity strengths. Notes: red represents IGE, and blue represents HC. **Fig. S6.** a. Correlation between binarized and non-binarized thalamo-cerebral FC profiles. b. Correlation between binarized and non-binarized thalamo-cerebellar FC profiles. c. Distribution of non-binarized thalamo-cerebral FC profiles under [50, 60] connectivity bin. d. Distribution of binarized thalamo-cerebral FC profiles under [50, 60] connectivity bin. e. Distribution of non-binarized thalamo-cerebellar FC profiles under [40, 50] connectivity bin. f. Distribution of binarized thalamo-cerebellar FC profiles under [40, 50] connectivity bin. Notes: red represents IGE, and blue represents HC. **Fig. S7.** a. Thalamo-cerebral FC profiles of HC at different connectivity strengths were obtained by averaging the thresholded and non-binarized FC matrix. b. Thalamo-cerebral FC profiles of IGE at different connectivity strengths were obtained by averaging the thresholded and non-binarized FC matrix. **Fig. S8.** a. Thalamo-cerebellar FC profiles of HC at different connectivity strengths were obtained by averaging the thresholded and non-binarized FC matrix. b. Thalamo- cerebellar FC profiles of IGE at different connectivity strengths were obtained by averaging the thresholded and non-binarized FC matrix. **Fig. S9.** The distribution of non-binarized thalamo-cerebral FC profiles across functional networks and group comparisons under varying connectivity strengths. Notes: * indicates uncorrected *p* < 0.05, ** indicates Bonferroni-corrected *p* < 0.05; red represents IGE, and blue represents HC. FC: functional connectivity; VN: visual network; SMN: sensorimotor network; LN: limbic network; DMN: default mode network, FPN: frontoparietal network; VAN: ventral attention network; DAN: dorsal attention network. **Fig. S10.** The distribution of non-binarized thalamo-cerebellar FC profiles across functional networks and group comparisons under varying connectivity strengths. Notes: * indicates uncorrected *p* < 0.05, ** indicates Bonferroni-corrected *p* < 0.05; red represents IGE, and blue represents HC. **Fig. S11.** a. Correlation analysis of thalamo-cerebral gradients derived from binarized and non-binarized FC. b. Correlation analysis of thalamo-cerebellar gradients derived from binarized and non-binarized FC. Notes: red represents IGE, and blue represents HC. **Fig. S12.** a. The thalamo-cerebral gradient distribution between groups was compared under varying connectivity strengths using Kolmogorov-Smirnov test. b. The thalamo-cerebellar gradient distribution between groups was compared under varying connectivity strengths using Kolmogorov-Smirnov test. Notes: * indicates Bonferroni-corrected *p* < 0.05; red represents the gradient distribution of IGE, and blue represents the gradient distribution of HC. **Fig. S13.** a. Correlation analysis of thalamo-cerebral eccentricity derived from binarized and non-binarized FC. b. Correlation analysis of thalamo-cerebellar eccentricity derived from binarized and non-binarized FC. Notes: red represents IGE, and blue represents HC. **Fig. S14.** a. Between-group difference in thalamo-cerebral eccentricity in IGE versus HC using non-binarized FC. b. Between-group difference in thalamo-cerebellar eccentricity in IGE versus HC using non-binarized FC. Notes: * indicates uncorrected *p* < 0.05, ** indicates FDR-corrected *p* < 0.05. **Fig. S15.** a. Correlation between thalamo-cerebral FC profiles under preprocessing conditions with and without GSR. b. Correlation between thalamo-cerebellar FC profiles under preprocessing conditions with and without GSR. Notes: red represents IGE, and blue represents HC. **Fig. S16.** a. Correlation between thalamo-cerebral gradients under preprocessing conditions with and without GSR. b. Correlation between thalamo-cerebellar gradients under preprocessing conditions with and without GSR. Notes: red represents IGE, and blue represents HC. **Fig. S17.** a. The thalamo-cerebral gradient distribution between groups was compared under varying connectivity strengths based on data processed without global signal regression. b. The thalamo-cerebellar gradient distribution between groups was compared under varying connectivity strengths based on data processed without global signal regression. Notes: * indicates Bonferroni-corrected *p* < 0.05; red represents the gradient distribution of IGE, and blue represents the gradient distribution of HC. **Fig. S18.** a. Correlation between thalamo-cerebral eccentricity under preprocessing conditions with and without GSR. b. Correlation between thalamo-cerebellar eccentricity under preprocessing conditions with and without GSR. Notes: red represents IGE, and blue represents HC. **Fig. S19.** a. Between-group difference in thalamo-cerebral eccentricity between IGE and HC (FDR; corrected *p* < 0.05) based on data processed without global signal regression. b. Between-group difference in thalamo-cerebellar eccentricity between IGE and HC based on data processed without global signal regression (FDR-corrected *p* < 0.05). **Fig. S20.** Between-group comparison of working memory performance between IGE patients and an independent sample of 133 age- and sex-matched HC. IGE patients showed significantly lower accuracy and longer reaction times compared to HC (two-sample t-tests; accuracy: t = -4.07, p = 6.62 × 10⁻⁵; reaction time: t = 4.24, *p* = 3.29 × 10⁻⁵). Data are presented as mean ± standard deviation. Accuracy is shown as percentage (%), and reaction time is shown in seconds (s). Red bars represent IGE, and blue bars represent HC. **Fig. S21.** a. Group difference in thalamo-cerebral eccentricity between IGE and HC, controlling for medication status, age, sex, and mFD (FDR-corrected *p* < 0.05). b. Group difference in thalamo-cerebellar eccentricity between IGE and HC, controlling for medication status, age, sex, and mFD (FDR-corrected *p* < 0.05). **Table S1.** Differences in network distribution of thalamo-cerebral FC profiles. **Table S2.** Differences in network distribution of thalamo-cerebellar FC. **Table S3.** The orientation of thalamo-cerebral gradient in IGE. **Table S4.** The orientation of thalamo-cerebellar gradient in IGE. **Table S5.** Difference in thalamo-cerebral eccentricity. **Table S6.** Difference in thalamo-cerebellar eccentricity.

## Data Availability

The data that support the findings of this study are available on reasonable request from the corresponding author.

## Abbreviations

AV: Anteroventral nucleus
CL: Central lateral nucleus
CM: Center median nucleus
CTC: Cerebellum-related thalamocortical pathway
DAN: Dorsal attention network
FC: Functional connectivity
fMRI: Functional magnetic resonance imaging
GSR: Global signal regression
HC: Healthy control
IGE: Idiopathic generalized epilepsy
LP: Lateral posterior nucleus
MDpc: Mediodorsal nucleus parvocellular part
PuM: Medial pulvinar
VAN: Ventral attention network
