# Supplementary material for: Disrupted thalamic functional hierarchy on resting-state fMRI in idiopathic generalized epilepsy
Source: Eur Radiol Exp. 2026 Sep 15;10:131. doi: 10.1186/s41747-026-00788-x (PMC13578145; doi:10.1186/s41747-026-00788-x)
Supplement: Supplementary file 1 [file 41747_2026_788_MOESM1_ESM.pdf]

Disrupted thalamic functional hierarchy on resting-state fMRI in idiopathic generalized epilepsy

ELECTRONIC SUPPLEMENTARY MATERIAL

I. Supplemental Methods

1 Image Data Acquisition

Magnetic resonance imaging (MRI) data were acquired using a 3T MRI scanner (Discovery MR750, GE). All participants were given foam padding to reduce head motion. The fMRI images were collected using an echo-planar imaging sequence. The scanning parameters were as follows: slices = 35; TR/TE = 2000 ms/30 ms; flip angle = 90°; FOV = 240×240 mm^2; matrix size = 64×64 and thickness = 4 mm; bandwidth = 250 kHz and 255 volumes in each run. Axial anatomical T1-weighted images were attained using a 3-dimensional fast spoiled gradient echo sequence: TR = 6.012 ms, TE = 1.968 ms, FA = 9°, matrix = 256×256, FOV = 256×256 mm^2, slice thickness = 1 mm, 152 slices without a gap. An 8-channel phased-array head coil was used for data acquisition. During resting-state fMRI scanning, the subjects were instructed to keep their eyes closed and not to fall asleep.

2 Data Preprocessing

Preprocessing of functional magnetic resonance imaging (fMRI) and T1-weighted images followed the DPARSF pipeline [1]. Structural images underwent skull stripping, reorientation, and coregistered to functional images, followed by DARTEL-based tissue segmentation and generation of deformation fields. Functional preprocessing included discarding the first five volumes, slice-timing and head-motion correction, and exclusion of participants with motion > 2 mm or 2°. Functional images were then normalized to Montreal Neurological Institute space with 2-mm isotropic resolution using the DARTEL deformation fields. Afterward, linear trends were removed, and

nuisance regression included the first five CompCor components, Friston-24 head-motion parameters, and global signal regression. To further mitigate motion effects, scrubbing was applied by removing volumes with framewise displacement > 0.5 mm, along with one preceding and one subsequent volume. Finally, temporal band-pass filtering (0.01-0.10 Hz) was performed. No spatial smoothing was performed because the functional gradient analysis was voxel-wise. To ensure the reliability of functional gradient analyses, strict data quality exclusion criteria were applied. Specifically:

1) Participants were required to retain more than 6 minutes of valid fMRI data after scrubbing, as shorter time series may yield unstable functional connectivity estimates and unreliable gradient representations [2].
2) Participants with insufficient cerebellar coverage in the registered fMRI or T1 images, as determined by visual inspection, were excluded. Complete cerebellar coverage is essential for accurately characterizing thalamo-cerebellar functional connectivity and gradient organization, as partial coverage can bias connectivity strength and spatial topology.
3) Participants with failed tissue segmentation, blurred images, or registration errors were excluded. Functional images were spatially normalized using DARTEL-based deformation fields [3], a method that has been widely applied in neuroimaging studies [4, 5]. Given that the present study focused on deep brain structures, including the thalamus and cerebellum, direct EPI-based normalization may provide suboptimal spatial accuracy in these regions. The DARTEL-based pipeline enables more precise alignment of deep and infratentorial structures. The DARTEL analysis relies on accurate gray and white matter segmentation of T1-weighted structural images, whereas blurring of T1 images can substantially compromise segmentation quality and subsequently impair the accuracy of spatial normalization. Because voxel-wise and region-wise functional connectivity analyses are highly sensitive to segmentation and registration accuracy, preprocessing errors may introduce spurious correlations and distort gradient embeddings. Therefore, data with such issues were strictly excluded.

3 Head Motion Quantification

The head motion was quantified following the approach proposed by Power [6], and the mean frame-wise displacement (mFD) of each subject was computed using the following formula:

$$\mathrm{mFD} = \left(\frac{1}{M-1}\right)\sum_{i=2}^{M}\left(\left|\Delta d_{x_i^1}\right| + \left|\Delta d_{y_i^1}\right| + \left|\Delta d_{z_i^1}\right| + \left|\Delta d_{x_i^2}\right| + \left|\Delta d_{y_i^2}\right| + \left|\Delta d_{z_i^2}\right|\right)$$

where *M* denotes the number of timepoints; x_i^1/x_i^2, y_i^1/y_i^2, and z_i^1/z_i^2 represent translations/rotations at the *i*th timepoint in the *x*, *y*, and *z* directions, respectively; and $\Delta d_{x_i^1} = x_i^1 - x_{i-1}^1$, and a similar pattern held for the others. Rotational parameters were converted from degrees to millimeters by projecting them onto the surface of a sphere with a radius of 50 mm.

4 Mapping of thalamic nuclei for clusters with intergroup differences

Following a previous study [7], each cluster showing significant intergroup differences was mapped to thalamic nuclei defined in the Morel atlas [8] to quantify its spatial distribution across thalamic subregions. Specifically, each cluster was extracted to create a mask, and anatomical masks of left and right thalamic nuclei were loaded. The overlap between each nucleus mask and the cluster mask was computed in voxel space. The proportion of overlapping voxels relative to the total voxels in the cluster was defined as the spatial occupancy, reflecting the cluster's relative distribution across nuclei. To reduce potential bias from nuclei with very few voxels or incidental overlap, only nuclei with occupancy greater than 10% were retained for result presentation and anatomical interpretation. This analysis aimed to provide anatomical localization rather than statistical inference. Thus, no significance testing was performed at the nucleus level. Results were calculated and reported separately for the left and right thalamus to avoid hemispheric confounding.

References

1. Yan CG, Wang XD, Zuo XN, Zang YF (2016) DPABI: Data Processing & Analysis for (Resting-State) Brain Imaging. Neuroinformatics 14:339–351. https://doi.org/10.1007/s12021-016-9299-4

2. Hong SJ, Xu T, Nikolaidis A et al (2020) Toward a connectivity gradient-based framework for reproducible biomarker discovery. Neuroimage 223:117322. https://doi.org/10.1016/j.neuroimage.2020.117322

3. Ashburner J (2007) A fast diffeomorphic image registration algorithm. Neuroimage 38:95–113. https://doi.org/10.1016/j.neuroimage.2007.07.007

4. Ruan L, Chen G, Yao M et al (2024) Brain functional gradient and structure features in adolescent and adult autism spectrum disorders. Hum Brain Mapp 45:e26792. https://doi.org/10.1002/hbm.26792

5. Rainer LJ, Kuchukhidze G, Trinka E et al (2023) Recognition and perception of emotions in juvenile myoclonic epilepsy. Epilepsia 64:3319–3330. https://doi.org/10.1111/epi.17783

6. Power JD, Barnes KA, Snyder AZ, Schlaggar BL, Petersen SE (2012) Spurious but systematic correlations in functional connectivity MRI networks arise from subject motion. Neuroimage 59:2142–2154. https://doi.org/10.1016/j.neuroimage.2011.10.018

7. Gong J, Luo C, Li X et al (2019) Evaluation of functional connectivity in subdivisions of the thalamus in schizophrenia. Br J Psychiatry 214:288–296. https://doi.org/10.1192/bjp.2018.299

8. Krauth A, Blanc R, Poveda A et al (2010) A mean three-dimensional atlas of the human thalamus: generation from multiple histological data. Neuroimage 49:2053–2062. https://doi.org/10.1016/j.neuroimage.2009.10.042

II. Supplemental Results

Robustness and sensitivity of functional gradients

functional gradients were more sensitive to global signal regression, with stability varying across connectivity bins and gradient dimensions (Supplementary Fig. S16). Specifically, for the thalamo-cerebral system, the principal and second gradients showed good consistency under all connectivity conditions ($r > 0.40$), whereas the third gradient exhibited lower correlations under intermediate connectivity conditions. For the thalamo-cerebellar system, the principal and second gradients maintained high similarity under the [0,10] and [90,100] bins ($r > 0.40$), but showed poor stability under intermediate connectivity conditions. The third thalamo-cerebellar gradient showed low correlations under anticorrelated conditions and under the [80,90] bin, but relatively high correlations in the remaining bins ($r > 0.50$).

Moreover, the inclusion or exclusion of GSR differentially affected the between-group differences in gradient distributions (Supplementary Fig. S17). For thalamo-cerebral gradients, group differences in the principal gradient disappeared under anticorrelate and some intermediate bins, but persisted under the [90,100] bin. The second gradient showed additional group differences under the [80,90] bin. The third gradient retained differences in correlated and anticorrelated bins, and exhibited even larger differences in intermediate bins. For thalamo-cerebellar gradients, the principal gradient displayed group differences under the [90,100] and [0,10] bins. The spatial patterns of differences in the second and third gradients were largely preserved, with additional differences emerging under intermediate bins.

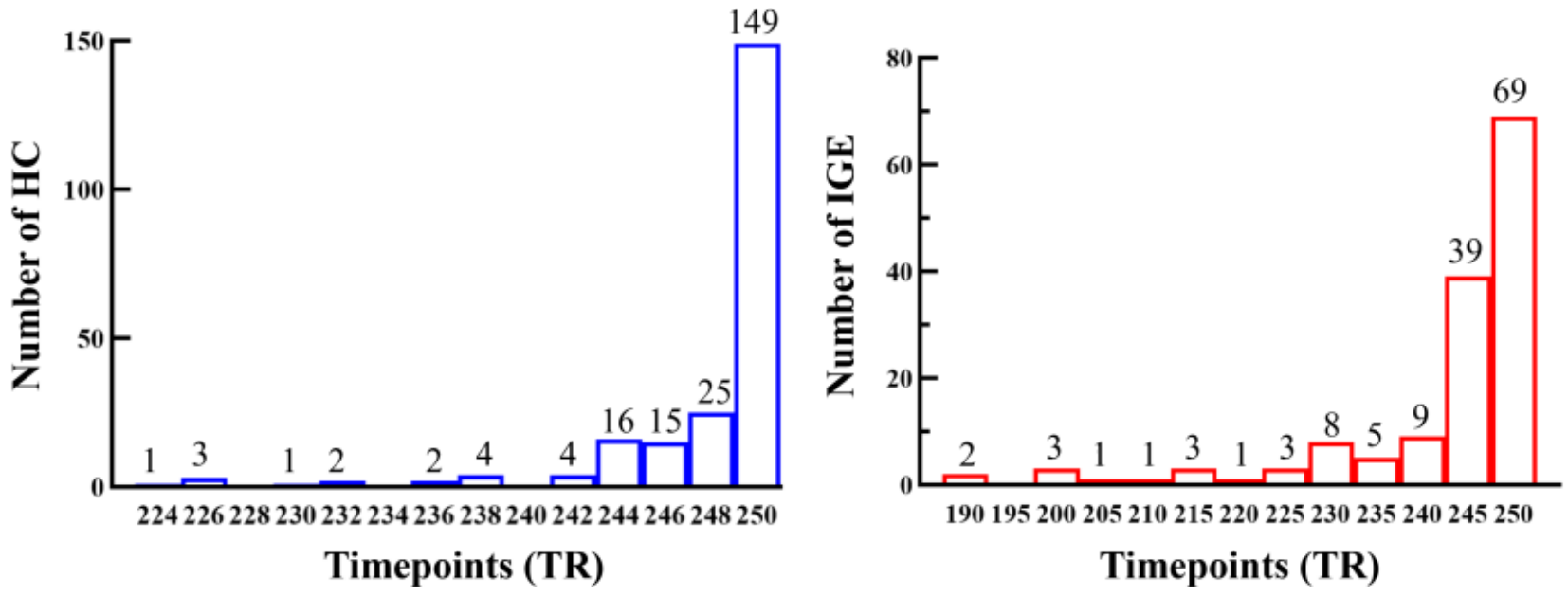

Fig. S1. Number of timepoints remaining after preprocessing for each participant. Notes: red represents IGE, and blue represents HC.

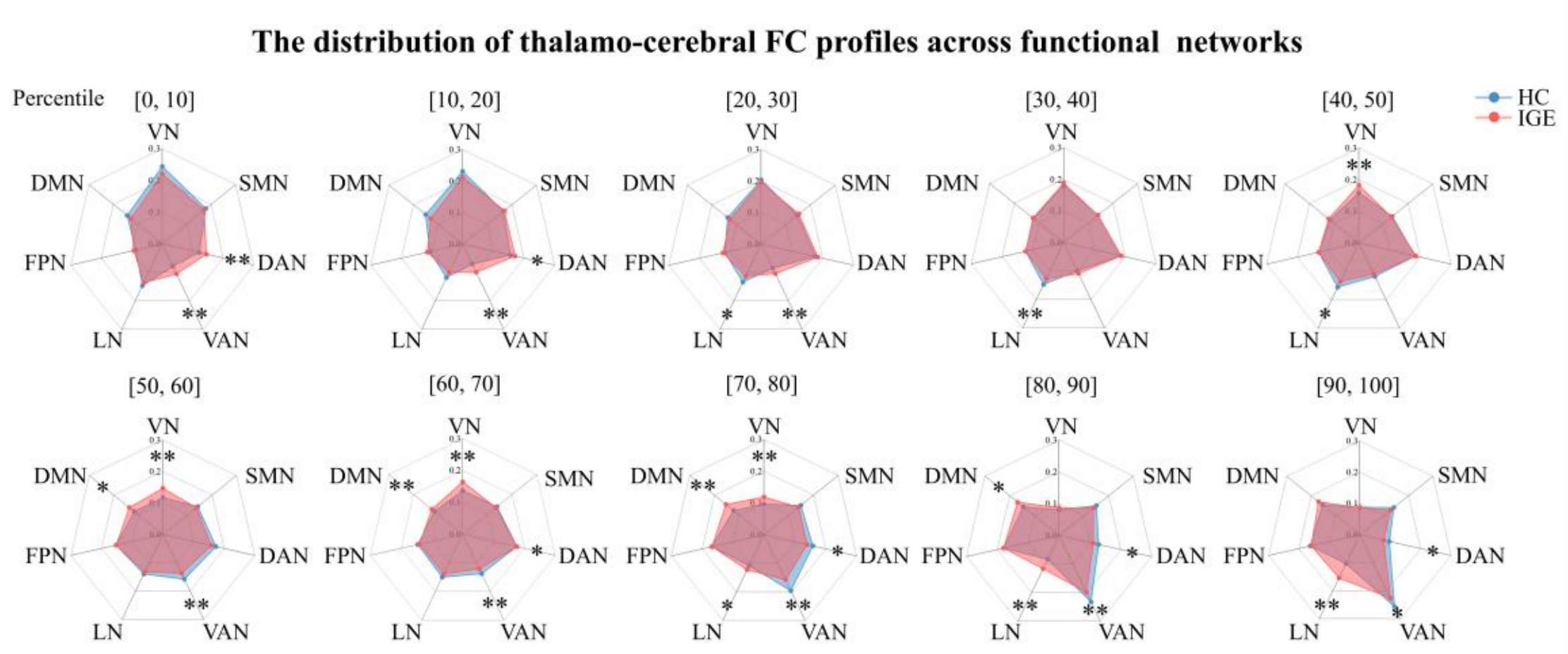

Fig. S2. Distribution of thalamo-cerebral FC profiles across networks and group comparisons under varying connectivity strengths. Notes: * indicates uncorrected $p < 0.05$, ** indicates Bonferroni-corrected $p < 0.05$; red represents IGE, and blue represents HC. Abbreviation: FC: functional connectivity; VN: visual network; SMN: sensorimotor network; LN: limbic network; DMN: default mode network, FPN: frontoparietal network; VAN: ventral attention network; DAN: dorsal attention network.

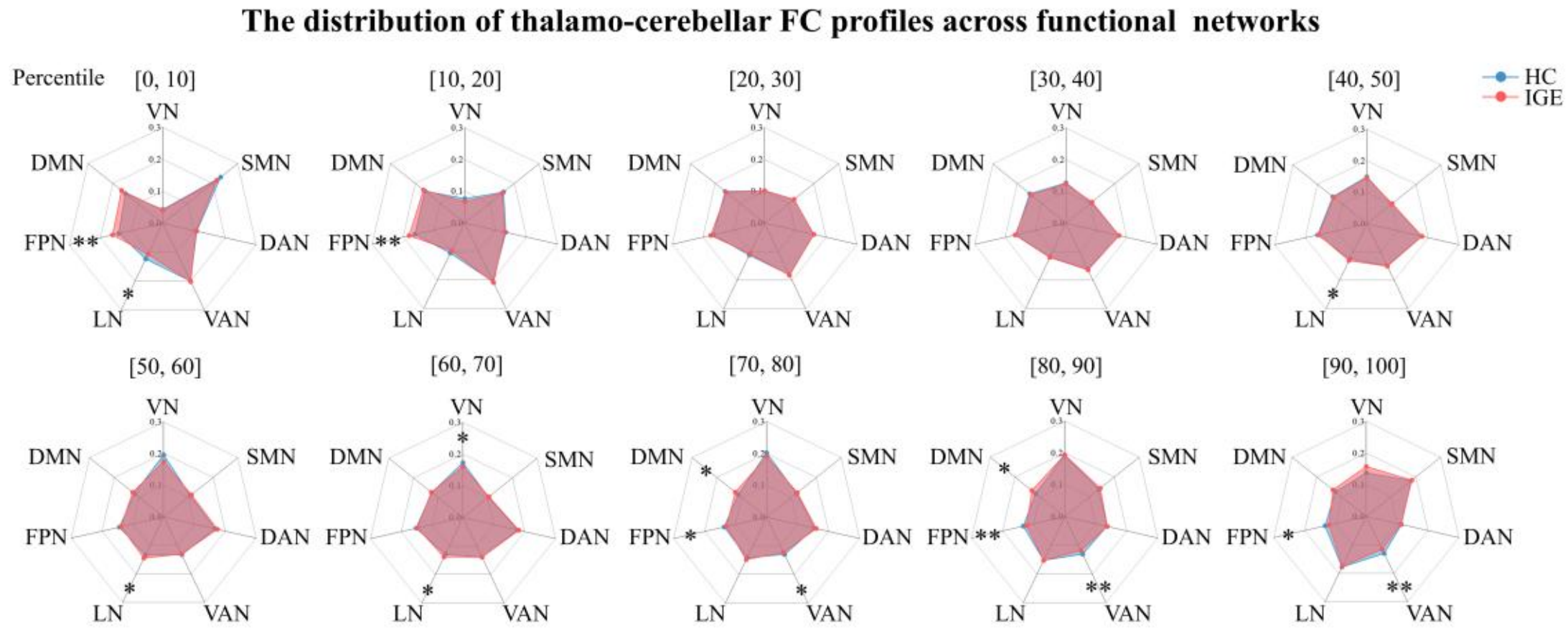

Fig. S3. Distribution of thalamo-cerebellar FC profiles across networks and group comparisons under varying connectivity strengths. Notes: * indicates uncorrected $p < 0.05$, ** indicates Bonferroni-corrected $p < 0.05$; red represents IGE, and blue represents HC. Abbreviation: FC: functional connectivity; VN: visual network; SMN: sensorimotor network; LN: limbic network; DMN: default mode network, FPN: frontoparietal network; VAN: ventral attention network; DAN: dorsal attention network.

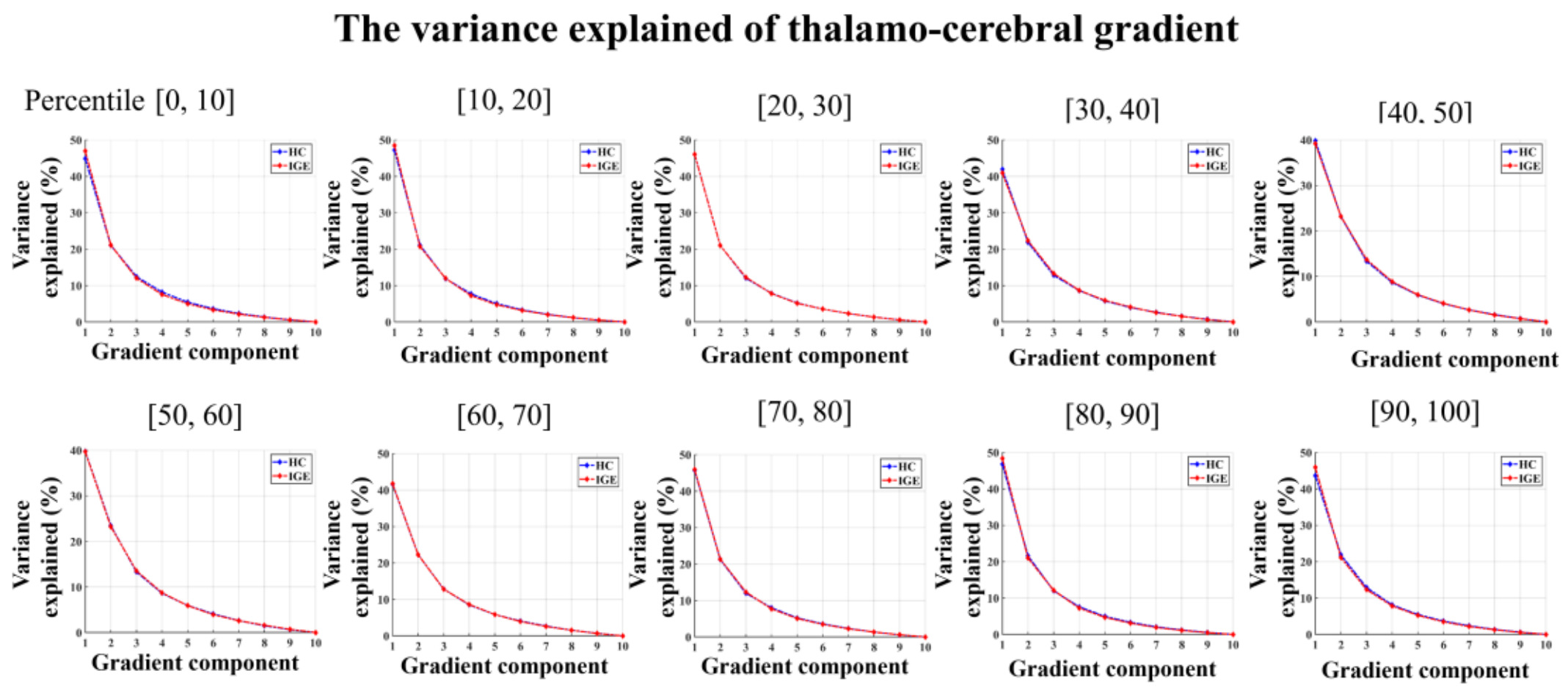

Fig. S4. Variance explained by thalamo-cerebral gradients across a full range of connectivity strengths. Notes: red represents IGE, and blue represents HC.

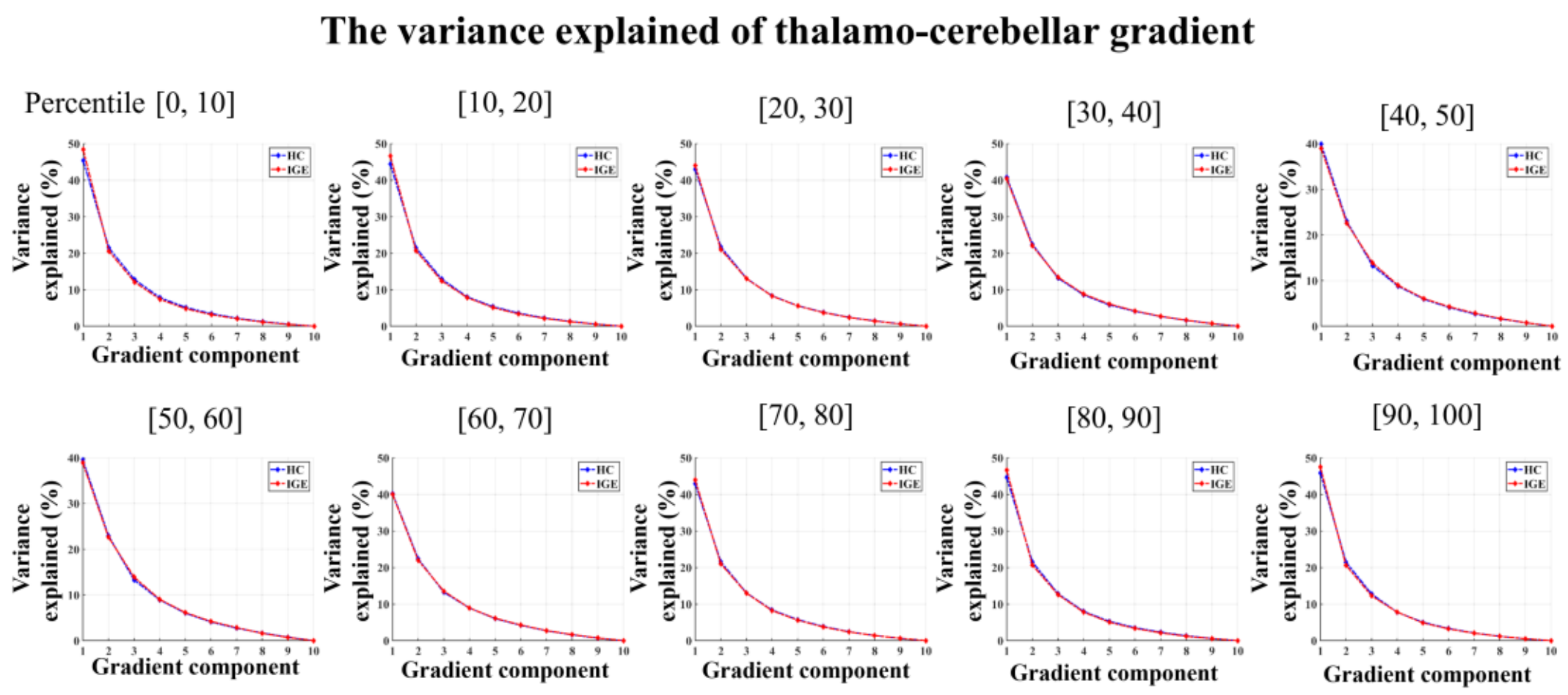

Fig. S5. Variance explained by thalamo-cerebellar gradients across a full range of connectivity strengths. Notes: red represents IGE, and blue represents HC.

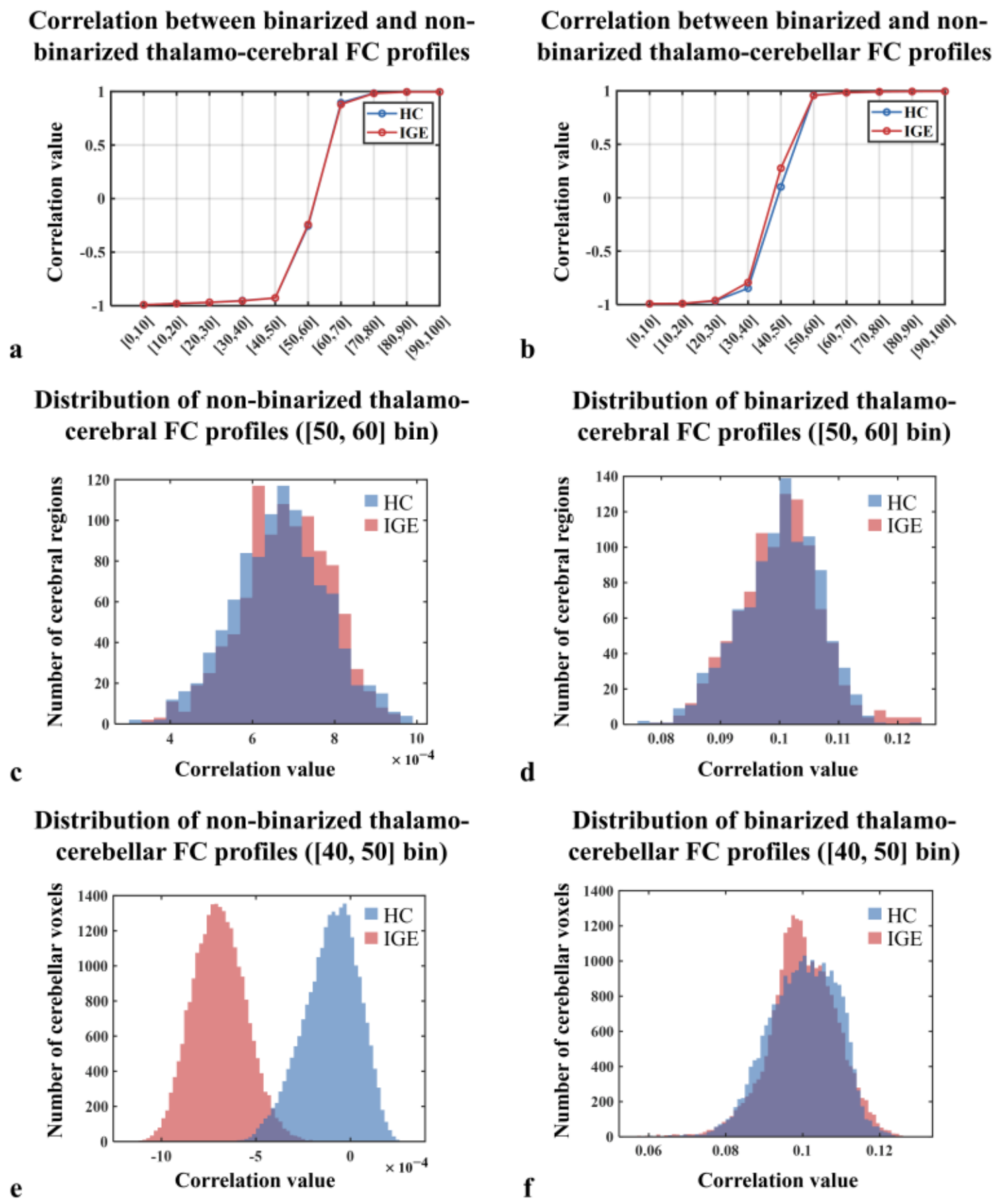

Fig. S6. a. Correlation between binarized and non-binarized thalamo-cerebral FC profiles. **b.** Correlation between binarized and non-binarized thalamo-cerebellar FC profiles. **c.** Distribution of non-binarized thalamo-cerebral FC profiles under [50, 60] connectivity bin. **d.** Distribution of binarized thalamo-cerebral FC profiles under [50, 60] connectivity bin. **e.** Distribution of non-binarized thalamo-cerebellar FC profiles under [40, 50] connectivity bin. **f.** Distribution of binarized thalamo-cerebellar FC profiles under [40, 50] connectivity bin. Notes: red represents IGE, and blue represents HC. Abbreviation: FC: functional

connectivity.

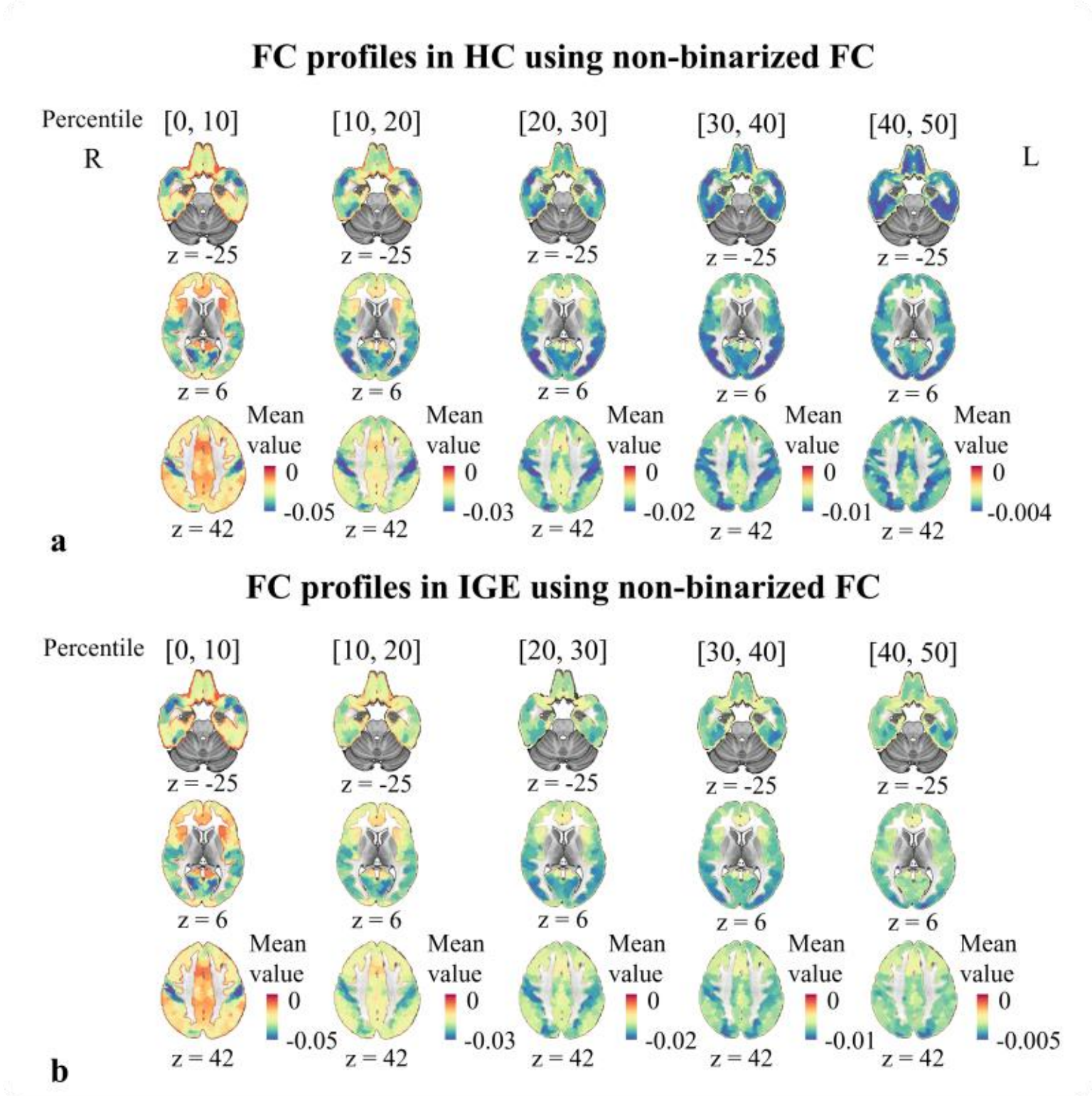

Fig. S7. a. Thalamo-cerebral FC profiles of HC at different connectivity strengths were obtained by averaging the thresholded and non-binarized FC matrix. **b.** Thalamo-cerebral FC profiles of IGE at different connectivity strengths were obtained by averaging the thresholded and non-binarized FC matrix; Abbreviation: FC: functional connectivity; R: right, L: left.

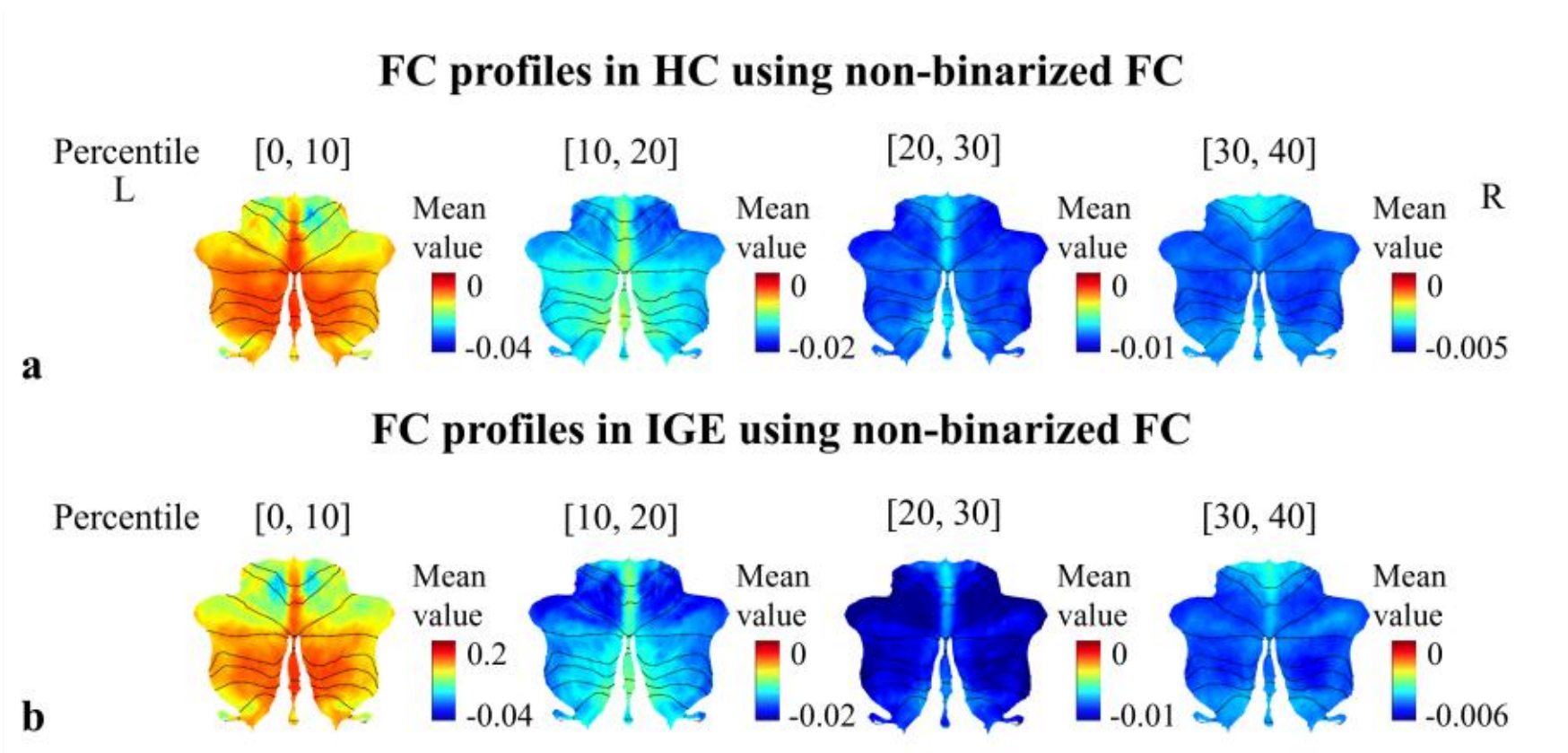

Fig. S8. a. Thalamo-cerebellar FC profiles of HC at different connectivity strengths were obtained by averaging the thresholded and non-binarized FC matrix. **b.** Thalamo-cerebellar FC profiles of IGE at different connectivity strengths were obtained by averaging the thresholded and non-binarized FC matrix; Abbreviation: FC: functional connectivity; R: right, L: left.

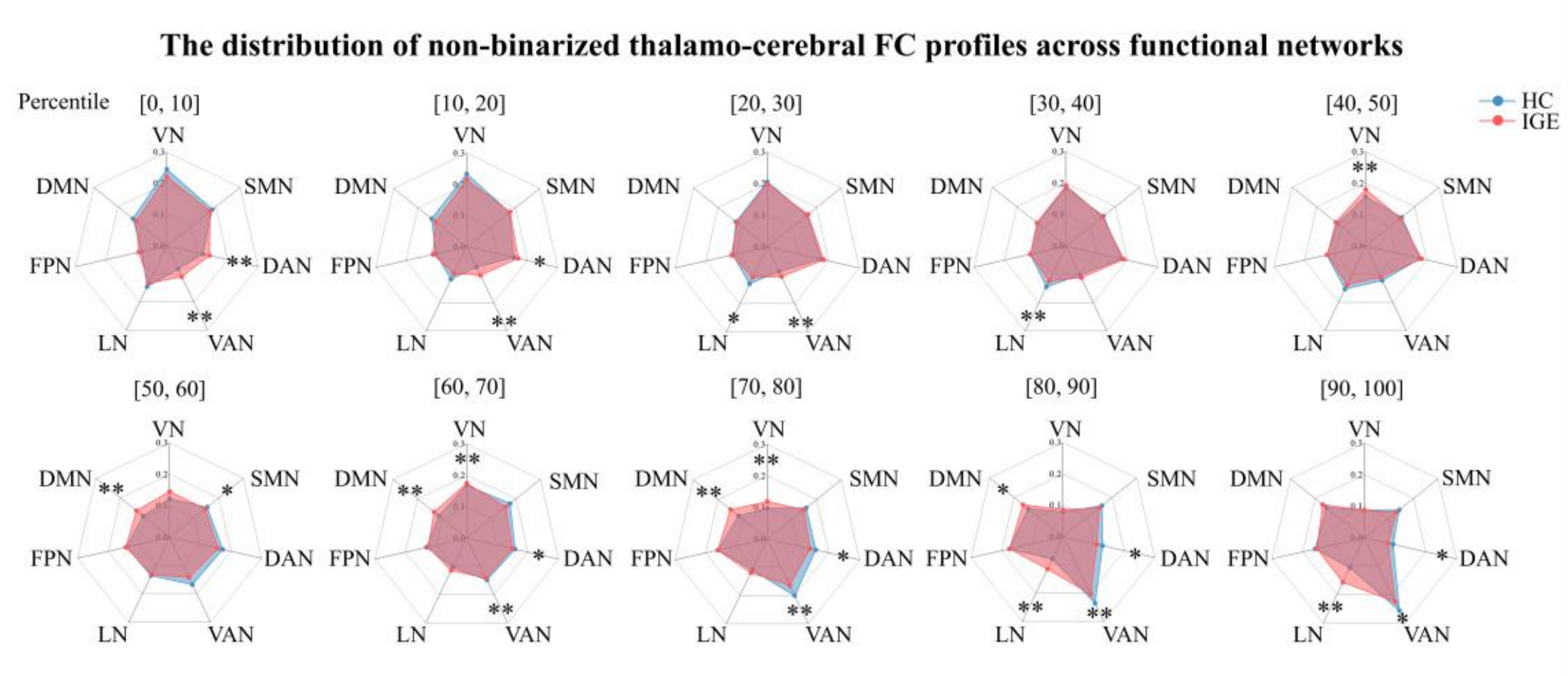

Fig. S9. The distribution of non-binarized thalamo-cerebral FC profiles across functional networks and group comparisons under varying connectivity strengths. Notes: * indicates uncorrected p < 0.05, ** indicates Bonferroni-corrected p < 0.05; red represents IGE, and blue represents HC. Abbreviation: FC: functional connectivity; VN: visual network; SMN: sensorimotor network; LN: limbic network; DMN: default mode network, FPN: frontoparietal network; VAN: ventral attention network; DAN: dorsal attention network.

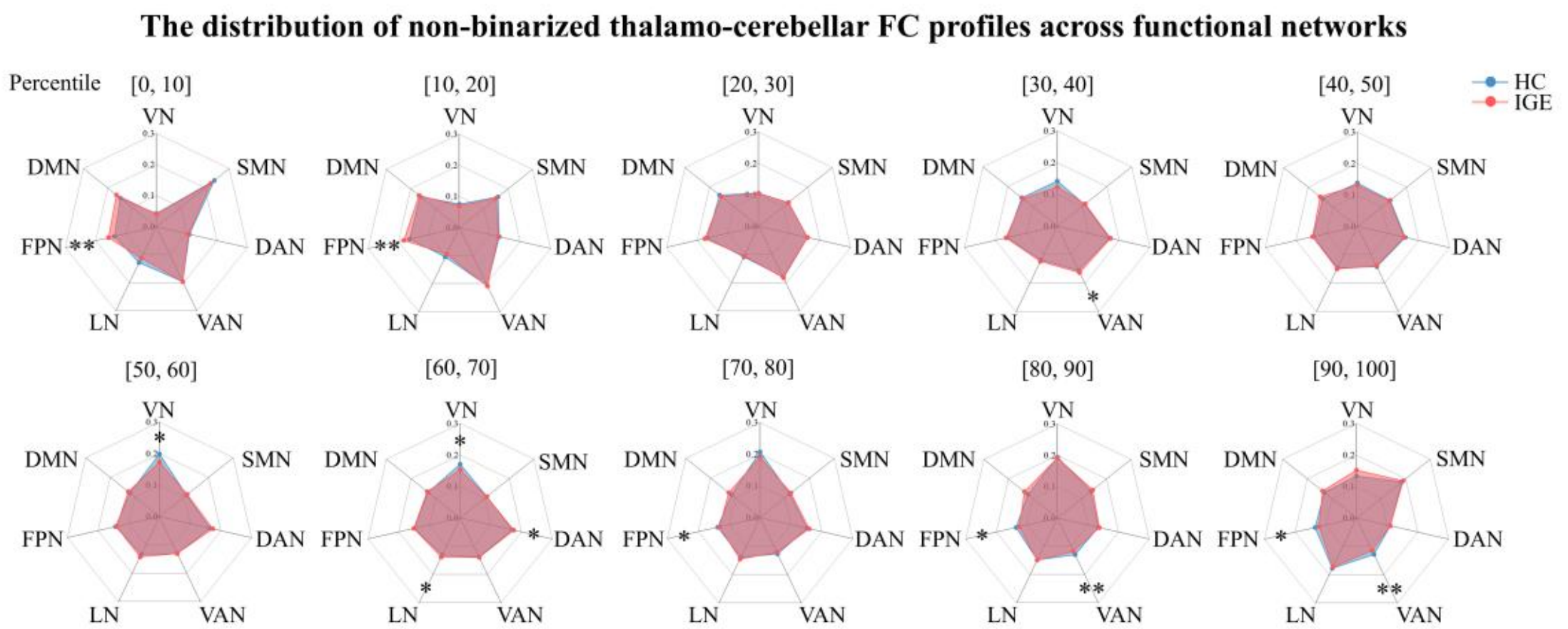

Fig. S10. The distribution of non-binarized thalamo-cerebellar FC profiles across functional networks and group comparisons under varying connectivity strengths. Notes: * indicates uncorrected p < 0.05, ** indicates Bonferroni-corrected p < 0.05; red represents IGE, and blue represents HC. Abbreviation: FC: functional connectivity; VN: visual network; SMN: sensorimotor network; LN: limbic network; DMN: default mode network, FPN: frontoparietal network; VAN: ventral attention network; DAN: dorsal attention network.

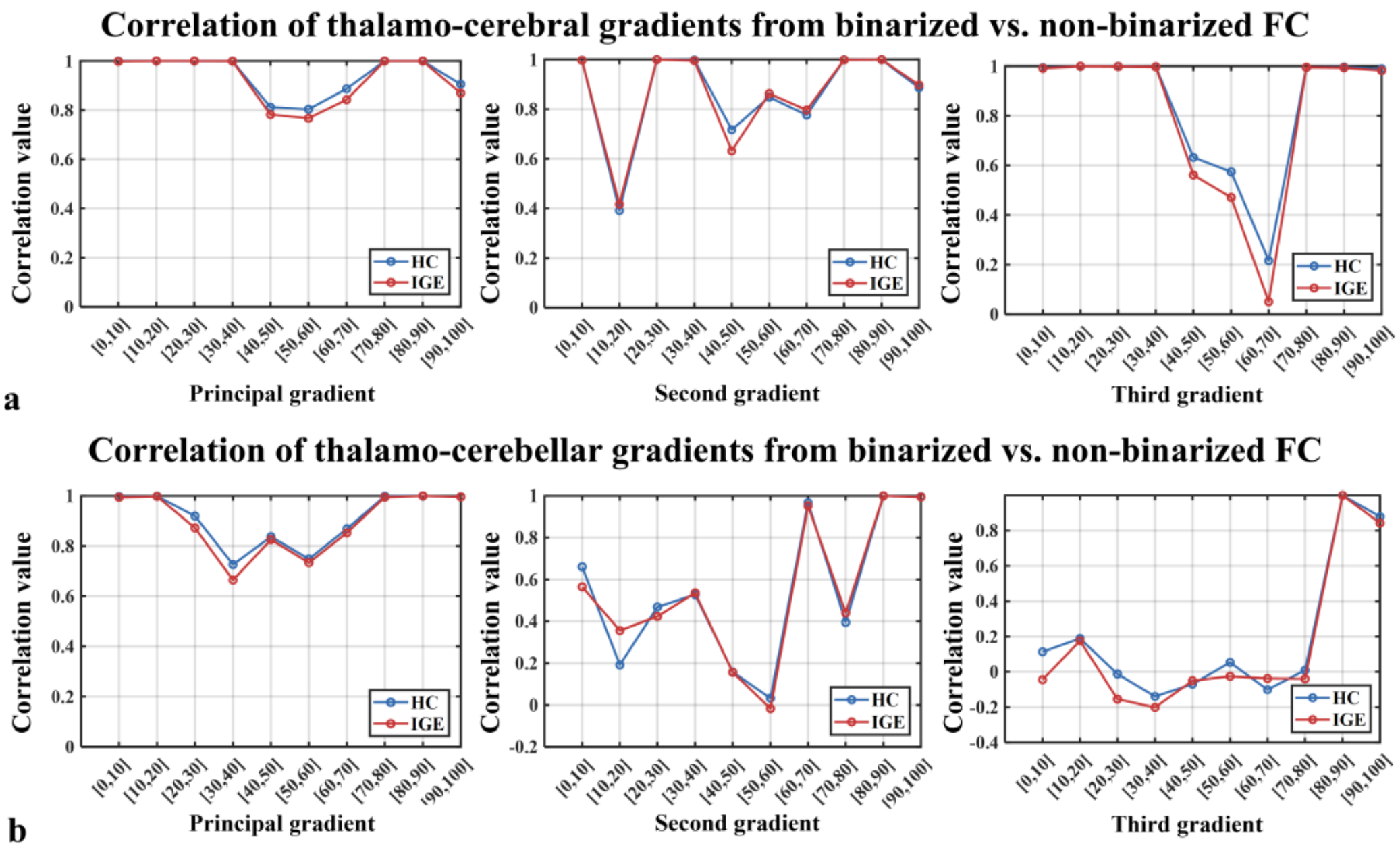

Fig. S11. a. Correlation analysis of thalamo-cerebral gradients derived from binarized and non-binarized FC. **b.** Correlation analysis of thalamo-cerebellar gradients derived from binarized and non-binarized FC. Notes: red represents IGE, and blue represents HC. Abbreviation: FC: functional connectivity.

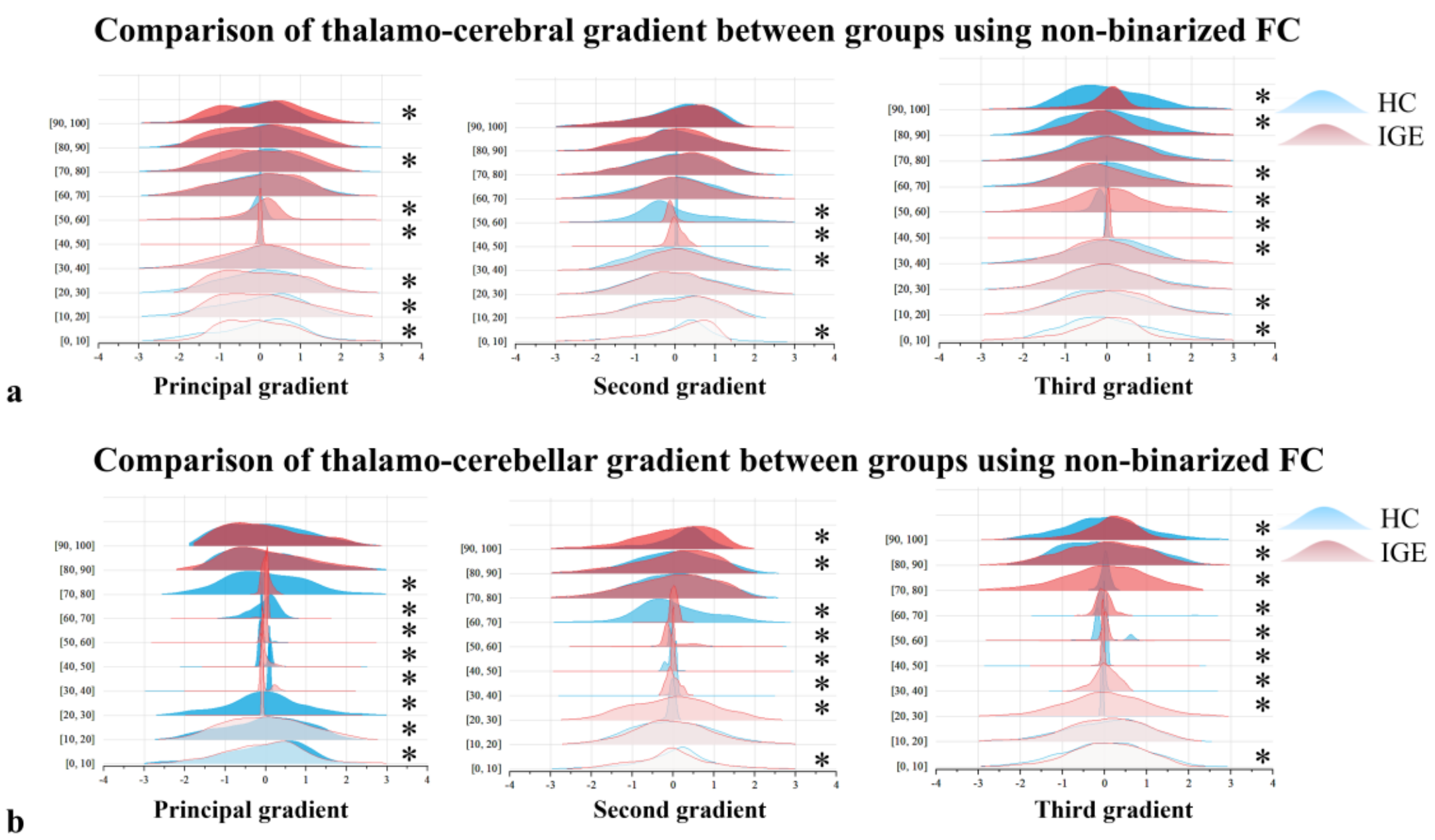

Fig. S12. a. The thalamo-cerebral gradient distribution between groups was compared under varying connectivity strengths using Kolmogorov-Smirnov test. **b.** The thalamo-cerebellar gradient distribution between groups was compared under varying connectivity strengths using Kolmogorov-Smirnov test. Notes: * indicates Bonferroni-corrected $p < 0.05$; red represents the gradient distribution of IGE, and blue represents the gradient distribution

of HC.

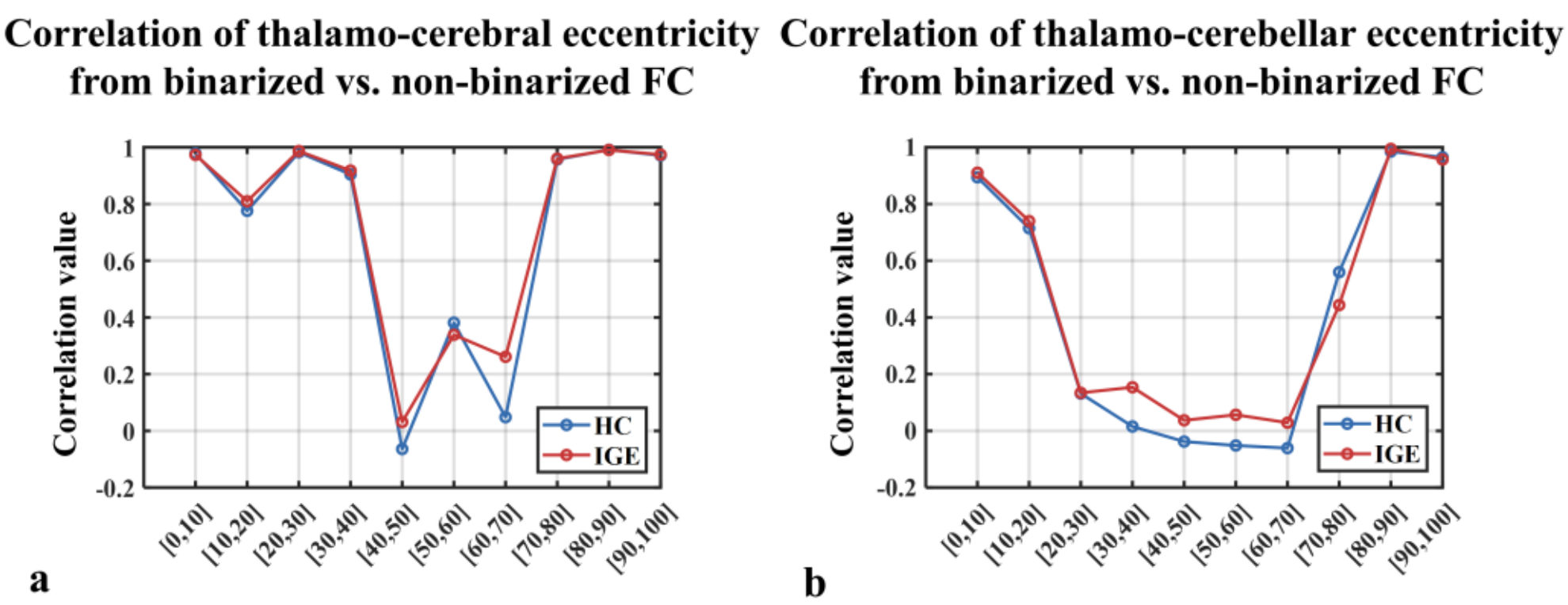

Fig. S13. a. Correlation analysis of thalamo-cerebral eccentricity derived from binarized and non-binarized FC. **b.** Correlation analysis of thalamo-cerebellar eccentricity derived from binarized and non-binarized FC. Notes: red represents IGE, and blue represents HC. Abbreviation: FC: functional connectivity.

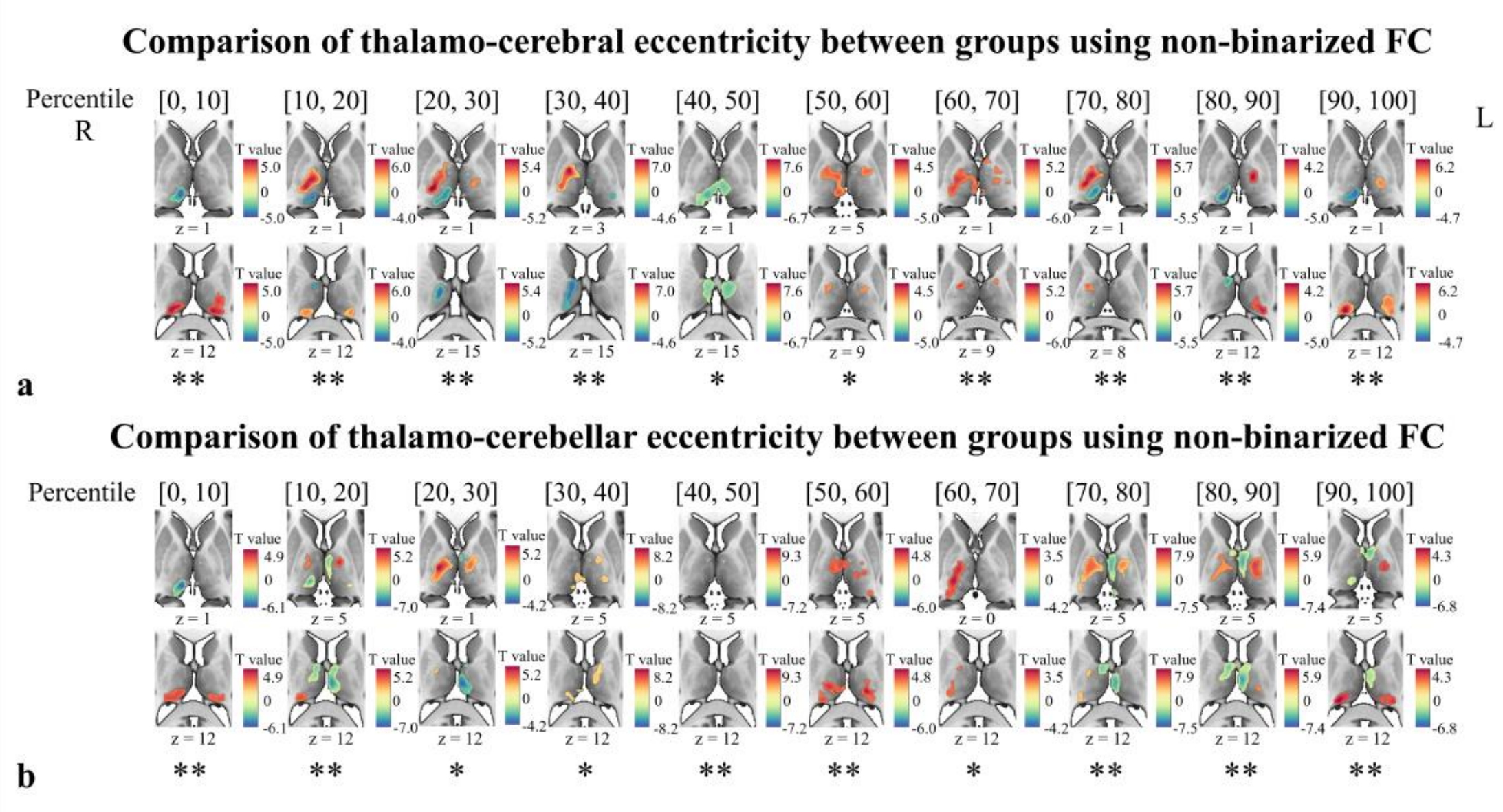

Fig. S14. a. Between-group difference in thalamo-cerebral eccentricity in IGE versus HC using non-binarized FC. **b.** Between-group difference in thalamo-cerebellar eccentricity in IGE versus HC using non-binarized FC. Notes: * indicates uncorrected p < 0.05, ** indicates FDR-corrected p < 0.05. Abbreviation: FC: functional connectivity; R: right, L: left.

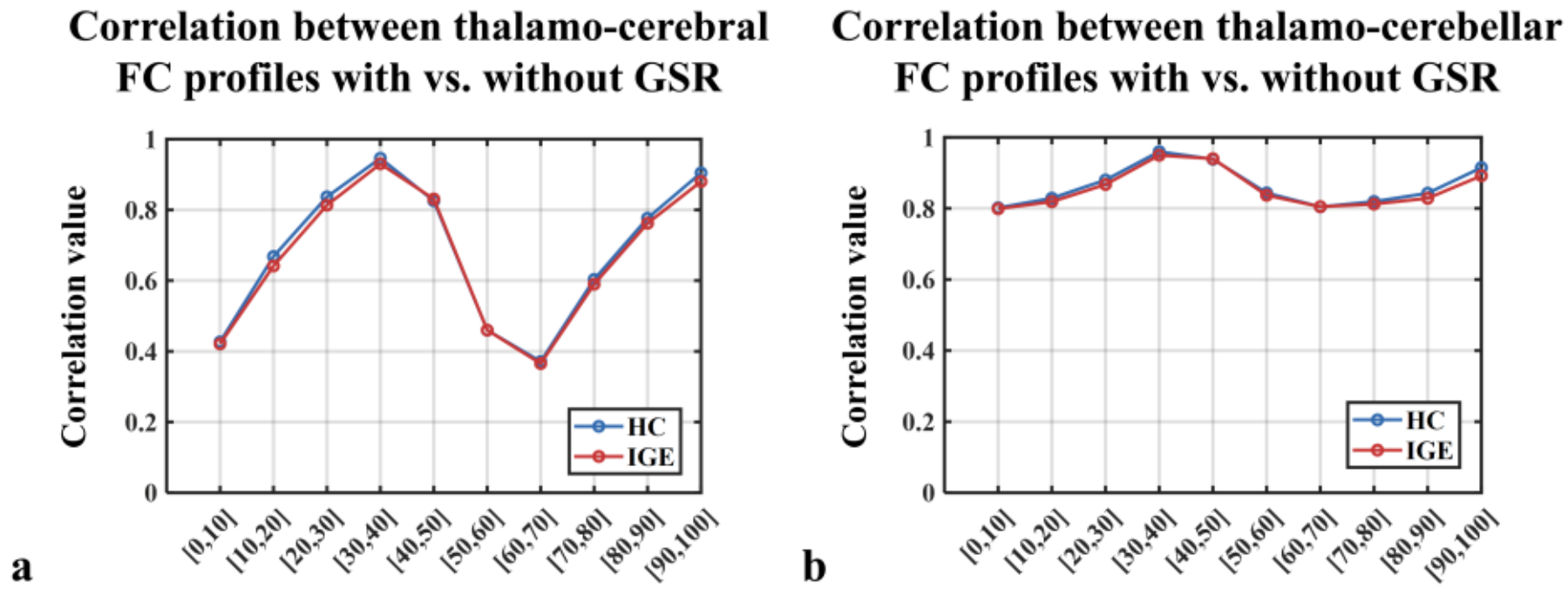

Fig. S15. a. Correlation between thalamo-cerebral FC profiles under preprocessing conditions with and without GSR. **b.** Correlation between thalamo-cerebellar FC profiles under preprocessing conditions with and without GSR. Notes: red represents IGE, and blue represents HC. Abbreviation: FC: functional connectivity; GSR: global signal regression.

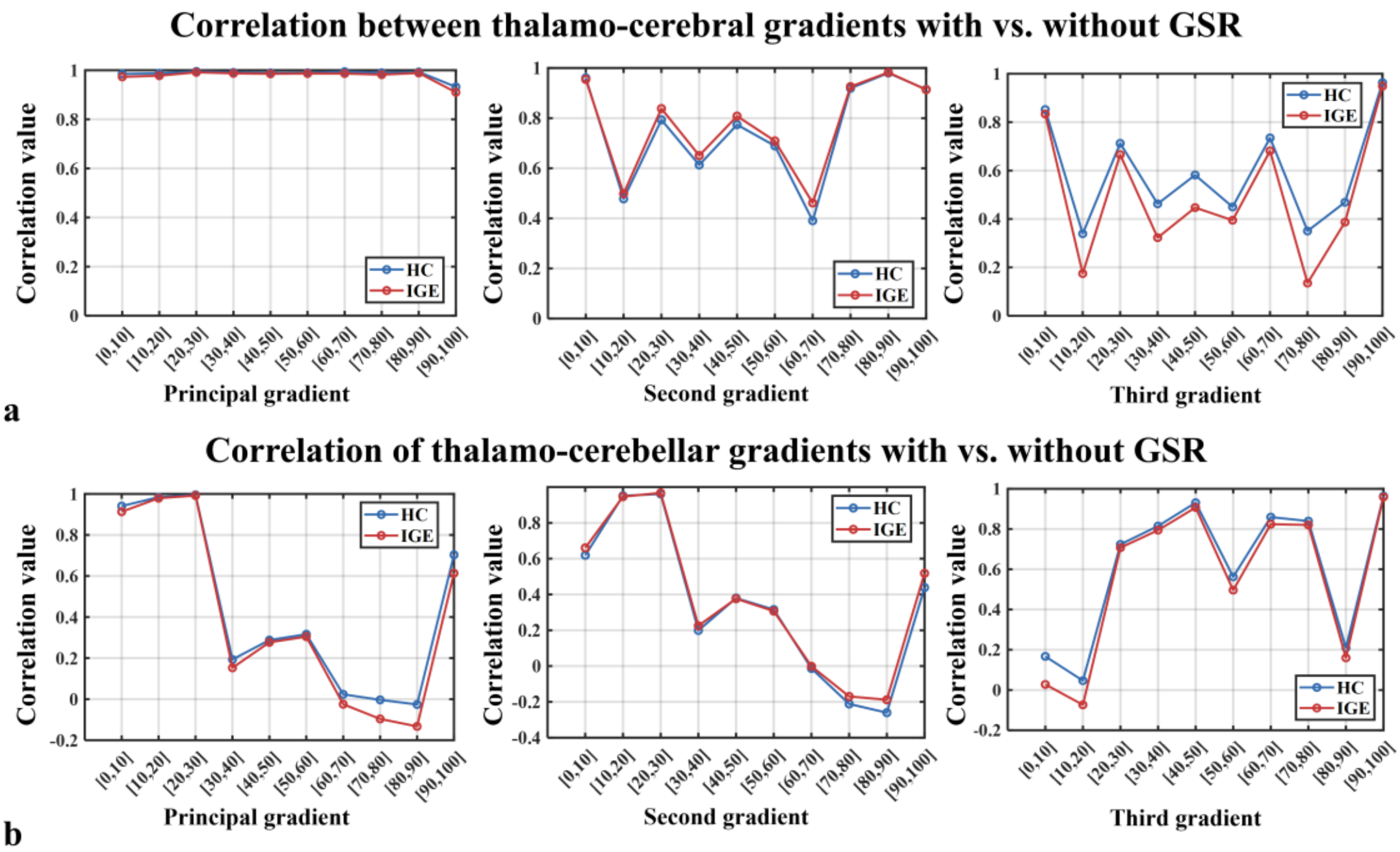

Fig. S16. a. Correlation between thalamo-cerebral gradients under preprocessing conditions with and without GSR. **b.** Correlation between thalamo-cerebellar gradients under preprocessing conditions with and without GSR. Notes: red represents IGE, and blue represents HC. Abbreviation: GSR: global signal regression.

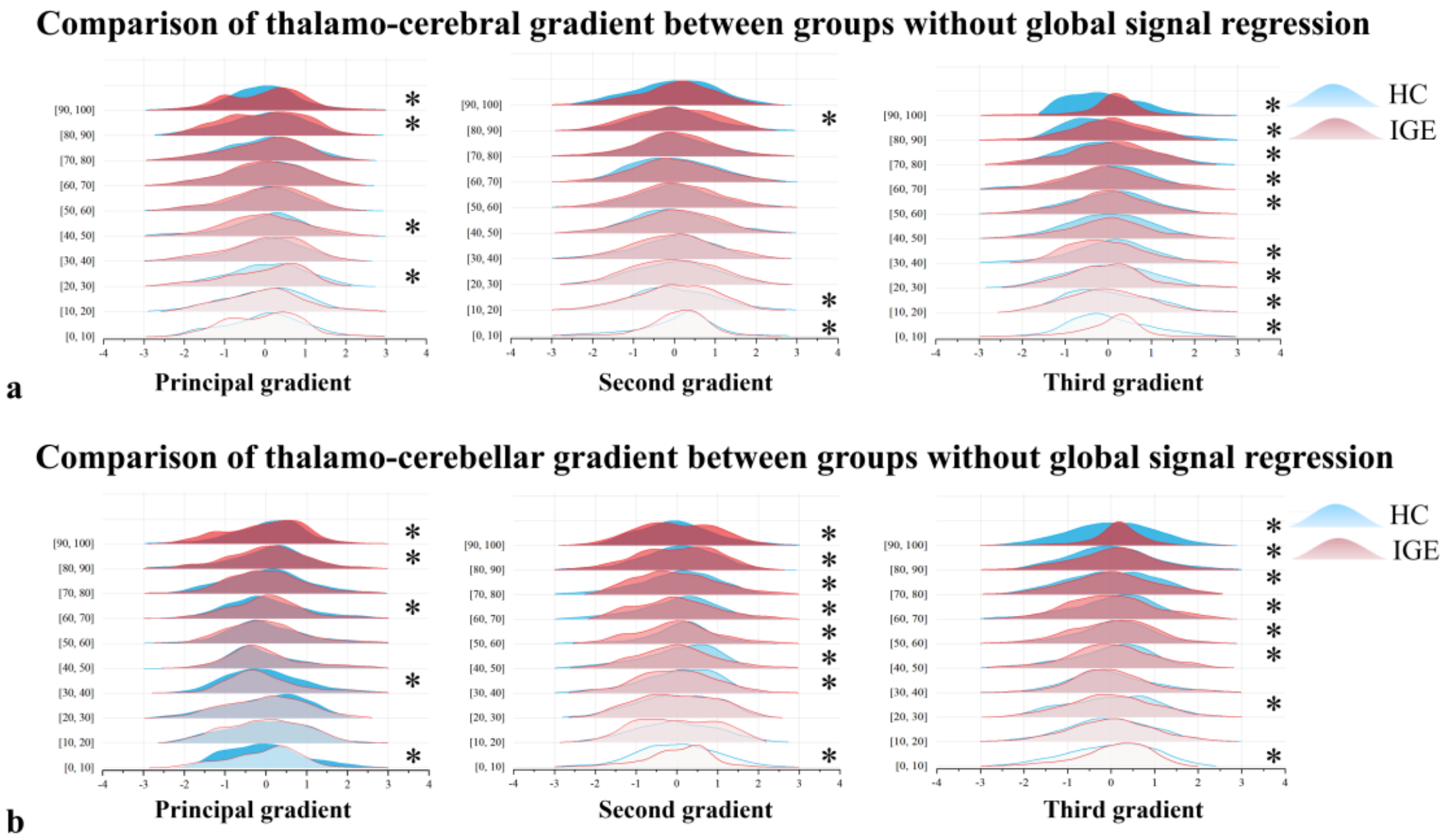

Fig. S17. a. The thalamo-cerebral gradient distribution between groups was compared under varying connectivity strengths based on data processed without global signal regression. **b.** The thalamo-cerebellar gradient distribution between groups was compared under varying connectivity strengths based on data processed without global signal regression. Notes: * indicates Bonferroni-corrected p < 0.05; red represents the gradient distribution of IGE, and blue represents the gradient distribution of HC.

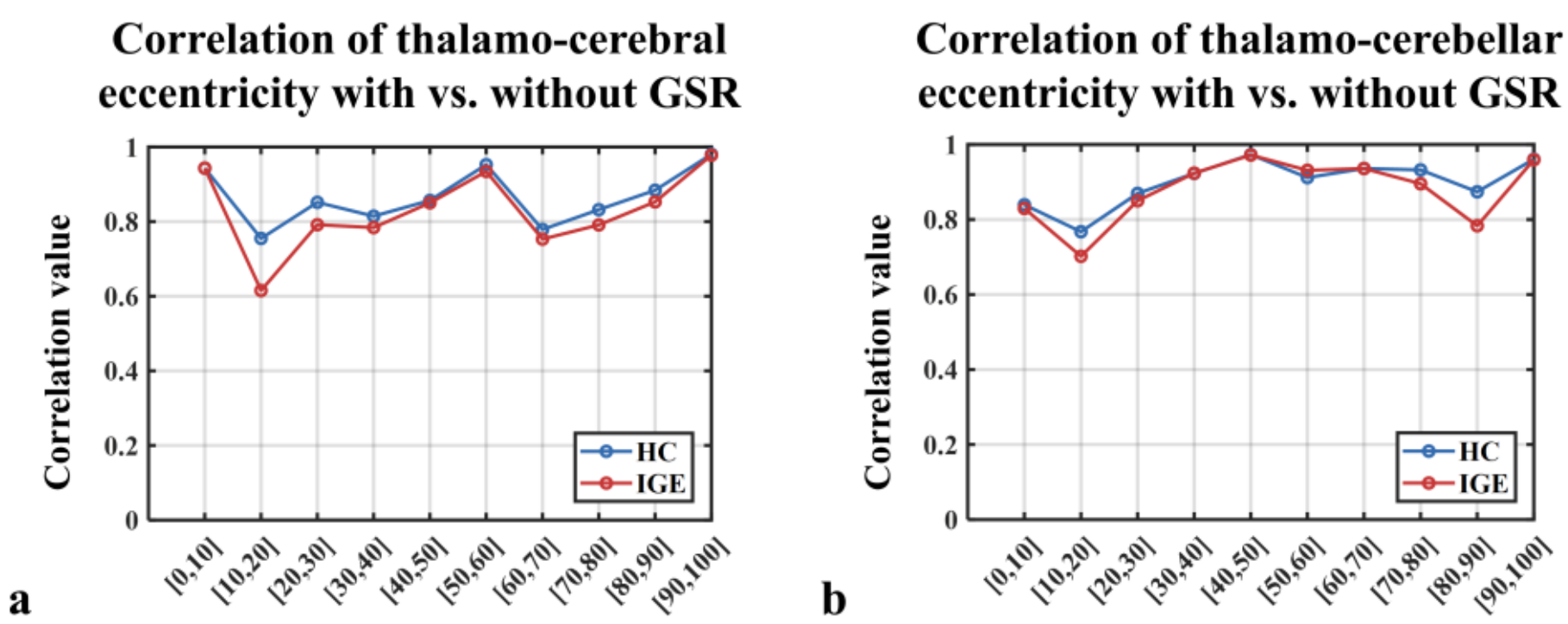

Fig. S18. a. Correlation between thalamo-cerebral eccentricity under preprocessing conditions with and without GSR. **b.** Correlation between thalamo-cerebellar eccentricity under preprocessing conditions with and without GSR. Notes: red represents IGE, and blue represents HC. Abbreviation: GSR: global signal regression.

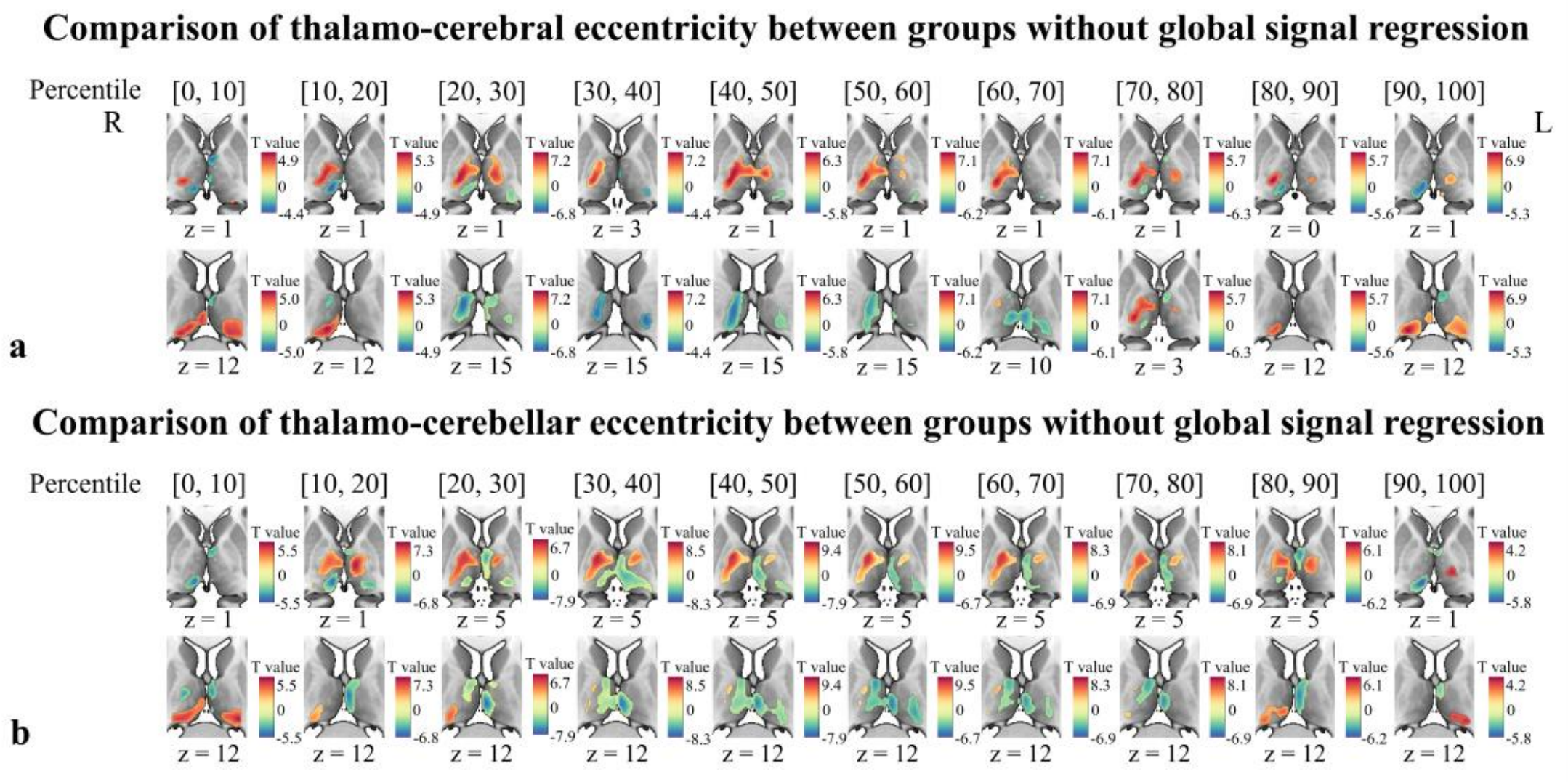

Fig. S19. a. Between-group difference in thalamo-cerebral eccentricity between IGE and HC (FDR; corrected $p < 0.05$) based on data processed without global signal regression. **b.** Between-group difference in thalamo-cerebellar eccentricity between IGE and HC based on data processed without global signal regression (FDR-corrected $p < 0.05$). Abbreviation: FDR: False Discovery Rate; R: right, L: left.

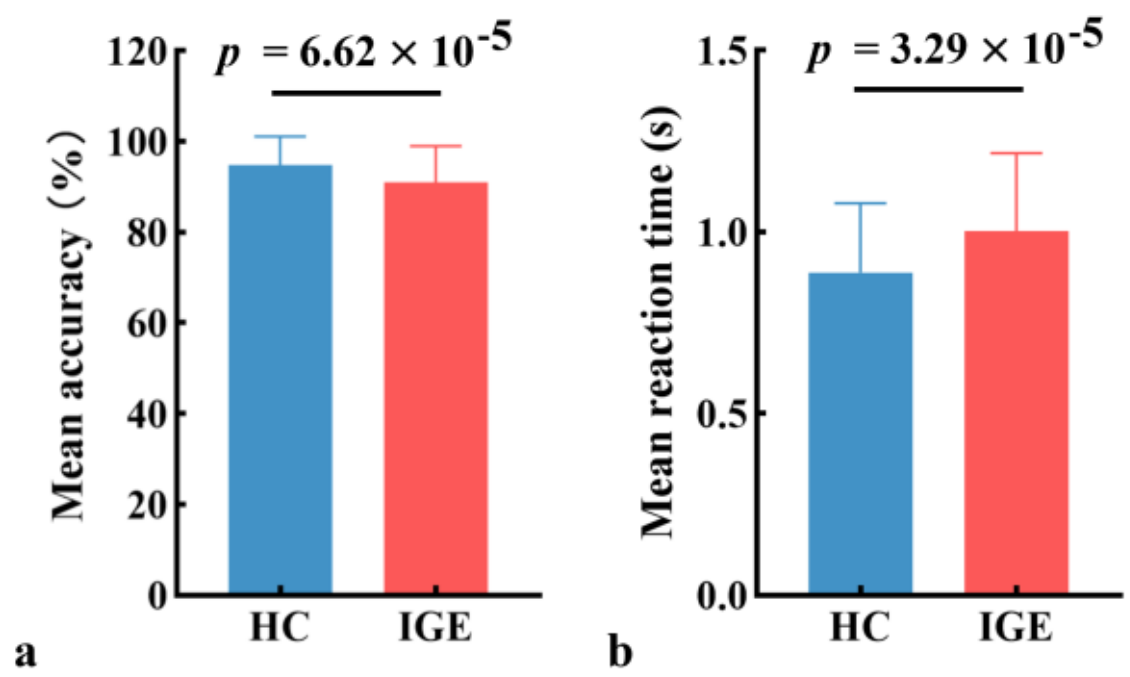

Fig. S20. Between-group comparison of working memory performance between IGE patients and an independent sample of 133 age- and sex-matched HC. IGE patients showed significantly lower accuracy and longer reaction times compared to HC (two-sample *t*-tests; accuracy: $t = -4.07$, $p = 6.62 \times 10^{-5}$; reaction time: $t = 4.24$, $p = 3.29 \times 10^{-5}$). Data are presented as mean ± standard deviation. Accuracy is shown as percentage (%), and reaction time is shown in seconds (s). Red bars represent IGE, and blue bars represent HC. Abbreviation: s, second.

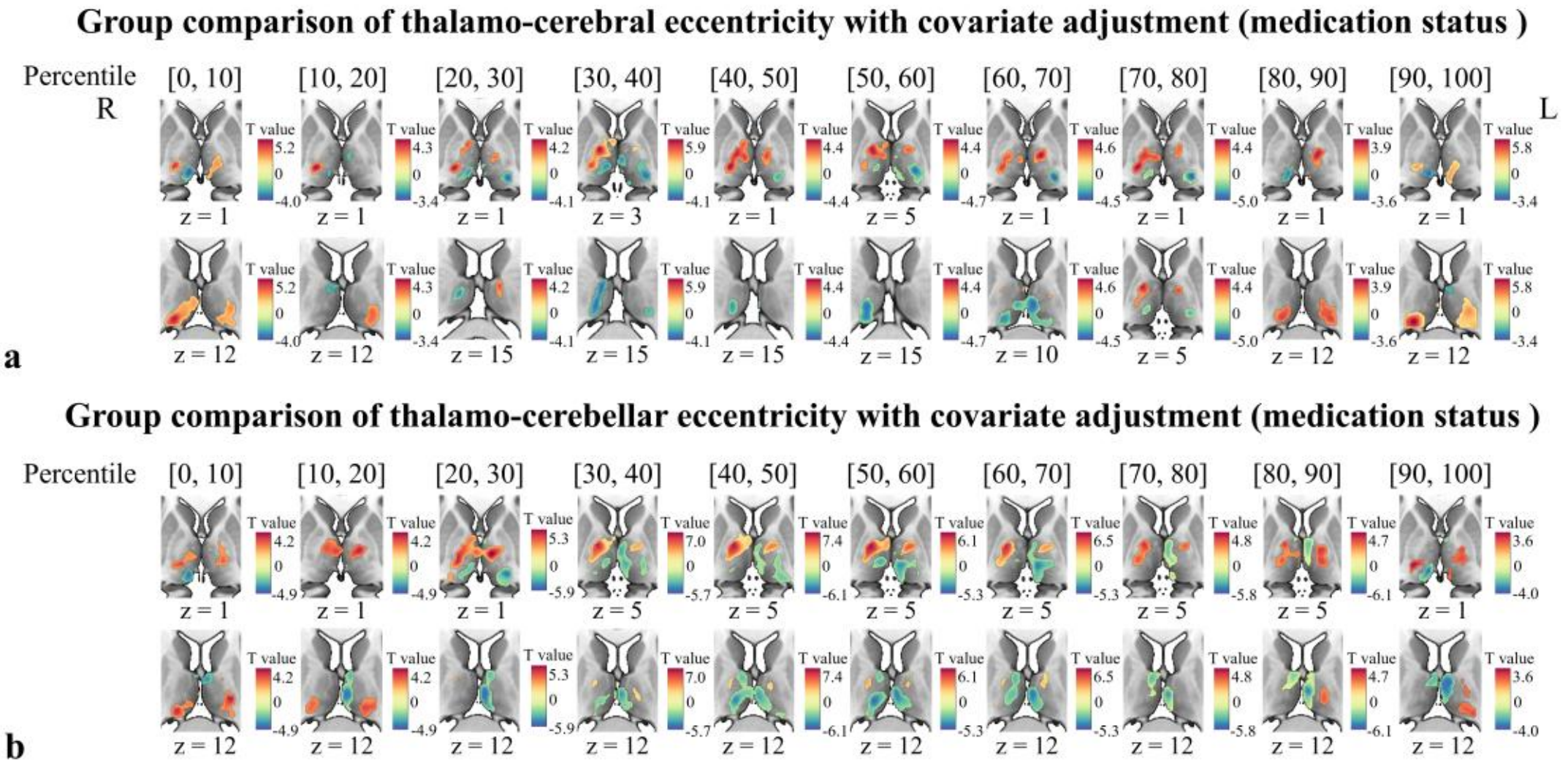

Fig. S21. a. Group difference in thalamo-cerebral eccentricity between IGE and HC, controlling for medication status, age, sex, and mFD (FDR-corrected p < 0.05). **b.** Group difference in thalamo-cerebellar eccentricity between IGE and HC, controlling for medication status, age, sex, and mFD (FDR-corrected p < 0.05). Abbreviation: FDR: False Discovery Rate; R: right, L: left; mFD: mean frame-wise displacement.

Table S1. Differences in network distribution of thalamo-cerebral FC profiles

Connectivity bins (percentile)	Network	*Z* value	*p* value
[0, 10]	DAN	3.08	0.002
	VAN	2.94	0.003
[10, 20]	DAN	2.47	0.014
	VAN	4.91	9.03×10^{-7}
[20, 30]	VAN	3.66	2.54×10^{-4}
	LN	-2.16	0.031
[30, 40]	LN	-2.95	0.003
[40, 50]	VN	3.09	0.002
	LN	-2.63	0.009
[50, 60]	VN	3.99	6.63×10^{-5}
	VAN	-3.63	2.88×10^{-4}
	DMN	2.24	0.025
[60, 70]	VN	4.09	4.40×10^{-5}
	DAN	-2.31	0.021
	VAN	-3.45	5.52×10^{-4}
	DMN	3.20	0.001
[70, 80]	VN	3.26	0.001
	DAN	-2.58	0.009
	VAN	-5.04	4.59×10^{-7}
	LN	2.00	0.045
	DMN	3.90	9.65×10^{-5}
[80, 90]	DAN	-2.30	0.021
	VAN	-2.87	0.004
	LN	4.06	4.95×10^{-5}
	DMN	2.41	0.016
[90, 100]	DAN	-2.33	0.019
	VAN	-2.44	0.015
	LN	5.10	3.31×10^{-7}

Abbreviation: FC: functional connectivity; VN: visual network; SMN: sensorimotor network; LN: limbic network; DMN: default mode network, FPN: frontoparietal network; VAN: ventral attention network; DAN: dorsal attention network.

Table S2. Differences in network distribution of thalamo-cerebellar FC profiles

Connectivity bins (percentile)	Network	*Z* value	*p* value
[0, 1]	LN	-2.00	0.045
	FPN	2.74	0.006
[10, 20]	FPN	3.60	3.23×10^{-4}
[40, 50]	LN	1.99	0.046
[50, 60]	LN	2.17	0.030
[60, 70]	VN	-2.18	0.029
	LN	2.46	0.014
[70, 80]	VAN	-2.44	0.015
	FPN	-2.44	0.015
	DMN	2.03	0.043
[80, 90]	VAN	-3.28	0.001
	FPN	-2.93	0.003
	DMN	2.14	0.033
[90, 100]	VAN	-3.99	6.52×10^{-5}
	FPN	-2.31	0.021

Abbreviation: FC: functional connectivity; VN: visual network; SMN: sensorimotor network; LN: limbic network; DMN: default mode network, FPN: frontoparietal network; VAN: ventral attention network; DAN: dorsal attention network.

Table S3. The orientation of thalamo-cerebral gradient in IGE

Connectivity bins (percentile)	Principal gradient	Second gradient	Third gradient
[0, 10]	Dorsomedial / ventrolateral axis	Anterior/ posterior axis	Similar to principal gradient, the dorsomedial part diverges into dorsoanterior and ventroposterior regions
[10, 20]	Dorsomedial / ventrolateral axis	Medial / lateral axis	Similar to principal gradient, the dorsoanterior part diverges into dorsoanterior and ventroposterior regions
[20, 30]	Dorsomedial / ventrolateral axis	Anterior/ posterior axis	Similar to principal gradient, the dorsomedial part diverges into dorsoanterior and ventroposterior regions
[30, 40]	Dorsoanterior / ventroposterior axis	Anterior/ posterior axis	Similar to principal gradient, the dorsomedial part diverges into dorsoanterior and ventroposterior regions
[40, 50]	Dorsoanterior / ventroposterior axis	Anterior/ posterior axis	Similar to principal gradient, the dorsomedial part diverges into dorsoanterior and ventroposterior regions
[50, 60]	Dorsoanterior / ventroposterior	Medial / lateral axis	Similar to principal gradient, the dorsomedial part diverges into dorsoanterior and ventroposterior regions
[60, 70]	Dorsoanterior / ventroposterior	Anterior/ posterior axis	Similar to principal gradient, the dorsomedial part diverges into dorsoanterior and ventroposterior regions
[70, 80]	Dorsoanterior / ventroposterior	Anterior/ posterior axis	Similar to principal gradient, the dorsomedial part diverges into dorsoanterior and ventroposterior regions
[80, 90]	Dorsomedial / ventrolateral axis	Anterior/ posterior axis	Similar to principal gradient, the dorsomedial part diverges into

			dorsoanterior and ventroposterior regions
[90, 100]	Dorsomedial / ventrolateral axis	Anterior/ posterior axis	Similar to principal gradient, the dorsomedial part diverges into dorsoanterior and ventroposterior regions

Table S4. The orientation of thalamo-cerebellar gradient in IGE

Connectivity bins (percentile)	Principal gradient	Second gradient	Third gradient
[0, 10]	Dorsomedial / ventrolateral axis	Anterior/ posterior axis	Anterolateral/ posteromedial axis
[10, 20]	Dorsomedial / ventrolateral axis	Anterior/ posterior axis	Dorsolateral / ventromedial axis
[20, 30]	Dorsomedial / ventrolateral axis	Anterior/ posterior axis	Similar to principal gradient, the ventrolateral part diverges into dorsomedial and ventrolateral regions
[30, 40]	Dorsomedial / ventrolateral axis	Anterior/ posterior axis	Anterolateral/ posteromedial axis
[40, 50]	Dorsomedial / ventrolateral axis	Anterior/ posterior axis	Anterolateral/ posteromedial axis
[50, 60]	Dorsomedial / ventrolateral axis	Anterior/ posterior axis	Anterolateral/ posteromedial axis
[60, 70]	Dorsomedial / ventrolateral axis	Anterior/ posterior axis	Anterolateral/ posteromedial axis
[70, 80]	Dorsomedial / ventrolateral axis	Anterior/ posterior axis	Anterolateral/ posteromedial axis
[80, 90]	Dorsomedial / ventrolateral axis	Anterior/ posterior axis	Anterolateral/ posteromedial axis
[90, 100]	Dorsomedial / ventrolateral axis	Anterior/ posterior axis	Similar to principal gradient, the ventrolateral part diverges into dorsomedial and ventrolateral regions

Table S5. Difference in thalamo-cerebral eccentricity

Connectivity bins (percentile)	Clusters	subregions	*T* value	Cluster size
[0, 10]	Cluster 1	right_CM/ right_Li/ right_Pf/ right_PuM	-5.40	92
	Cluster 2	left_LP/ left_PuM/ left_VPLp	4.40	57
	Cluster 3	right_CL/ right_LP/ right_PuM	5.37	90
[10, 20]	Cluster 1	right_CM/ right_PuM	-4.84	62
	Cluster 2	right_VPM	4.24	40
	Cluster 3	right_CL/ left_MDpc	-4.89	69
	Cluster 4	right_AV/ right_VLpd	-4.25	76
	Cluster 5	left_LP	3.97	27
[20, 30]	Cluster 1	right_CM/ right_MDpc/ right_Pf/ right_PuM	-4.60	80
	Cluster 2	left_VLa/ left_VLp/ left_VM	5.14	102
	Cluster 3	right_VApc/ right_VLa/ right_VLpv/ right_VM/ right_VPM	5.43	139
	Cluster 4	right_AV/ right_VLpd	-5.21	76
[30, 40]	Cluster 1	left_VM/ left_VPM	5.82	58
	Cluster 2	right_CL/ right_CM/ right_LP/ right_PuM/ right_VLpd/ right_VPLp	-5.16	165
	Cluster 3	right_VApc/ right_VLa/ right_VLpv/ right_VM/ right_VPLp/ right_VPM	7.72	217
	Cluster 4	left_LP/ left_PuL/ left_PuM/ left_VPLp	-4.98	116
	Cluster 5	left_CL/ left_MDmc/ left_MDpc	-5.76	84
[40, 50]	Cluster 1	left_CM/ left_Pf/ left_VLpv/ left_VM/ left_VPM	5.64	91

	Cluster 2	right_CL/ right_CeM/ right_MDpc/ right_VApc/ right_VLa/ right_VLpv/ right_VM/ right_VPLp/ right_VPM	7.69	302
	Cluster 3	left_CL/ left_MDmc/ left_MDpc/ left_PuM	-6.76	268
	Cluster 4	right_CL/ right_LP/ right_PuM/ right_VLpd	-4.58	133
[50, 60]	Cluster 1	left_VM/ right_MDpc/ right_VApc/ right_VLa/ right_VLpv/ right_VM/ left_VPLp	5.80	272
	Cluster 2	left_LP/ left_PuL/ left_PuM/ left_VPLp	-4.87	84
	Cluster 3	right_CL/ right_LP/ right_VLpd	-5.46	114
	Cluster 4	left_CL/ left_MDpc/ left_PuM	-4.81	124
[60, 70]	Cluster 1	left_VLa/ left_VLpd/ left_VM	4.73	84
	Cluster 2	right_MDpc/ right_VApc/ right_VLa/ right_VLpv/ right_VM/ right_VPLp/ right_VPM	5.96	189
	Cluster 3	left_CL/ left_MDmc/ left_MDpc/ left_PuL/ left_PuM/ right_CL/ right_LP/ right_MDpc/ right_PuM	-6.27	355
[70, 80]	Cluster 1	right_CM/ right_PuM	-5.57	98
	Cluster 2	right_VApc/ right_VLpv/ right_VLa/ right_VM/ right_VPM	5.71	156
[80, 90]	Cluster 1	left_LP/ left_VLa/ left_VLpv/ left_VM/ left_VPLp	4.55	139
	Cluster 2	right_CM/ right_Pf/ right_PuM	-5.06	93
	Cluster 3	right_AV/ right_VLpd	-4.76	59
[90, 100]	Cluster 1	right_CM/ right_Li/ right_Pf/ right_PuM	-5.33	94
	Cluster 2	left_CL/ left_LP/ left_PuM/ left_VLpv/ left_VPLp/ left_VPM	5.35	182
	Cluster 3	right_LD/ right_LP/ right_PuL/ right_PuM	6.38	106

Abbreviations: MDpc: Mediodorsal nucleus parvocellular part; MDmc: Mediodorsal nucleus

magnocellular part; CL: Central lateral nucleus; CeM: Central medial nucleus; CM: Centre median nucleus; Pf: Parafascicular nucleus; PuM: Medial pulvinar; PuL: Lateral pulvinar; PuA: Anterior pulvinar; LP: Lateral posterior nucleus; SG: Suprageniculate nucleus; Li: Limitans nucleus; Po: Posterior nucleus; VPLp: Lateral geniculate nucleus posterior part; VLa: Ventral lateral anterior nucleus; VLpd: Ventral lateral dorsal part; VLpv: Ventral lateral ventral part; VApc: Ventral anterior nucleus parvocellular part; VM: Ventral medial nucleus; AM: Anterior medial nucleus; AV: Anterior ventral nucleus; LD: Lateral dorsal nucleus; VPM: Ventral posterior medial nucleus.

Table S6. Difference in thalamo-cerebellar eccentricity

Connectivity bins (percentile)	Clusters	subregions	*T* value	Cluster size
[0, 10]	Cluster 1	right_Li/ right_PuM	-6.25	79
	Cluster 2	left_LP	3.98	19
	Cluster 3	right_LP/ right_PuM	5.01	46
[10, 20]	Cluster 1	right_PuM	-5.34	110
	Cluster 2	left_AV/ left_CL/ left_LD/ left_MDpc/ left_PuL/ left_puM/ left_VApc/ left_VLpd	-7.05	363
	Cluster 3	right_CeM/ right_MDpc/ right_MDmc/ right_VApc/ right_VLa/ right_VLpv/ right_VM/ right_VPM	4.94	221
	Cluster 4	left_VLa/ left_VLpv/ left_VM	5.28	78
[20, 30]	Cluster 1	left_AV/ left_CL/ left_LD/ left_MDmc/ left_MDpc/ left_PuL/ left_PuM/ left_VApc/ left_VLpd/ eight_AV/ left_VLpd	-7.75	466
	Cluster 2	right_CL/ right_CM/ right_Li/ right_MDpc/ right_PuA/ right_PuM	-5.48	135
	Cluster 3	left_VLa/ left_VLpv/ left_VM/ left_VPM/ right_CeM/ right_MDpc/ right_PuL/ right_VApc/ right_VLa/ right_VLpv/ right_VM/ right_VPLp/ right_VPM	7.20	429
[30, 40]	Cluster 1	left_CL/ left_MDpc/ left_MDmc/ left_PuM/ right_AV/ right_CL/ right_LP/ right_MDpc/ right_PuM/ right_VLpd	-8.27	543
	Cluster 2	left_CM/ left_VApc/ left_VLa/ left_VLpv/ left_VM/ left_VPM	6.39	138
	Cluster 3	right_CeM/ right_MDpc/ right_VApc/ right_VLa/ right_VLpv/ right_VM/ right_VPLp/ right_VPM	8.35	288
[40, 50]	Cluster 1	left_AV/ left_CL/ left_LD/ left_LP/ left_MDmc/ left_MDpc/ left_PuL/ left_PuM/ right_AV/right_CL/ right_LD/ right_LP/ right_MDmc/ right_MDpc/ right_VLpd/ right_VPLp	-7.24	577
	Cluster 2	left_CM/ left_VApc/ left_VLa/ left_VLpv/ left_VM/ left_VPM	6.47	157
	Cluster 3	right_CeM/ right_MDpc/ right_VApc/	9.30	318

		right_VLa/ right_VLpv/ right_VM/ right_VPLp/ right_VPM		
[50, 60]	Cluster 1	left_CL/ left_LD/ left_LP/ left_MDmc/ left_MDpc/ left_PuA/ left_PuM/ left_VLpd/ left_VPLp/ right_AV/ right_CL/ right_LD/ right_LP/ right_MDpc/ right_PuM/ right_VLpd/ right_VPLp	-6.95	682
	Cluster 2	left_VApc/ left_VLa/ left_VLpv/ left_VM/ left_VPM/ right_CeM/ right_MDpc/ right_PuL/ right_VApc/ right_VLa/ right_VLpv/ right_VM/ right_VPLp/ right_VPM	9.68	488
[60, 70]	Cluster 1	left_CL// left_MDpc/ left_MDmc/ left_PuA/ left_PuM/ left_VPLp	-6.91	283
	Cluster 2	right_AV/ right_CL/ right_LP/ right_MDpc/ right_VApc/ right_VLpd/ right_VPLp	-7.16	211
	Cluster 3	left_VApc/ left_VLpv/ left_VM/ right_CeM/ right_LP/ right_LP/ right_MDpc/ right_PuL/ right_PuM/ right_VApc/ right_VLa/ right_VLpd/ right_VLpv/ right_VM/ right_VPLp/ right_VPM	8.16	501
[70, 80]	Cluster 1	right_AV/ right_CL/ right_LP/ right_VLpd	-5.97	134
	Cluster 2	right_CeM/ right_MDpc/ right_VApc/ right_VLa/ right_VLpv/ right_VM/ right_VPLp/ right_VPM	7.96	258
	Cluster 3	left_VLa/ left_VM	4.29	84
	Cluster 4	left_CL/ left_MDpc/ left_MDmc/ left_PuM	-7.5	216
[80, 90]	Cluster 1	right_CeM/ right_MDpc/ right_VApc/ right_VLa/ right_VLpc/ right_VM/ right_VPM	4.77	192
	Cluster 2	left_PuM/ left_VLa/ left_VLpv/ left_VM/ left_VPLa/ left_VPLp/ left_VPM	5.34	187
	Cluster 3	left_AV/ left_CL/ left_MDpc/ left_VApc/ left_VLpd	-7.46	256
	Cluster 4	Right_AV/ right_VLpd	-5.28	96
[90, 100]	Cluster 1	right_CM/ right_Li/ right_Pf/ right_PuM	-6.34	104

Cluster 2	left_AV/ left_CL/ left_MDpc/ left_VApc/ left_VLpd	-5.22	137
Cluster 3	left_LP/ left_PuM	4.44	38

Abbreviations: MDpc: Mediodorsal nucleus parvocellular part; MDmc: Mediodorsal nucleus magnocellular part; CL: Central lateral nucleus; CeM: Central medial nucleus; CM: Centre median nucleus; Pf: Parafascicular nucleus; PuM: Medial pulvinar; PuL: Lateral pulvinar; PuA: Anterior pulvinar; LP: Lateral posterior nucleus; SG: Suprageniculate nucleus; Li: Limitans nucleus; Po: Posterior nucleus; VPLp: Lateral geniculate nucleus posterior part; VLa: Ventral lateral anterior nucleus; VLpd: Ventral lateral dorsal part; VLpv: Ventral lateral ventral part; VApc: Ventral anterior nucleus parvocellular part; VM: Ventral medial nucleus; AM: Anterior medial nucleus; AV: Anterior ventral nucleus; LD: Lateral dorsal nucleus; VPM: Ventral posterior medial nucleus.
